# An enhanced neural network algorithm and its applications for numerical optimization and parameter extraction of photovoltaic models

**DOI:** 10.1038/s41598-026-37918-9

**Published:** 2026-02-04

**Authors:** Aining Chi, Seyedali Mirjalili, Yiying Zhang

**Affiliations:** 1 School of Economics and Management, Jiangsu University of Science and Technology, Zhenjiang, 212003 China; 2 Centre for Artificial Intelligence Research and Optimization, Torrens University Australia, Brisbane, Australia; 3 Faculty of Electrical Engineering and Computer Science, VSB-Technical University of Ostrava, Ostrava, Czech Republic; 4 University Research and Innovation Center, Obuda University, Budapest, 1034 Hungary; 5 College of Electrical Engineering and Automation, Shandong University of Science and Technology, Qingdao, 266590 China

**Keywords:** Artificial neural network, Neural network algorithm, Parameter identification, Photovoltaic models, Global optimization, Computational biology and bioinformatics, Engineering, Health care, Mathematics and computing

## Abstract

Solar energy is a clean energy source with great application prospects. Photovoltaic (PV) system plays a very important role in converting solar energy into electricity. Optimizing, controlling, and simulating the PV system is of great significance for improving the conversion efficiency of solar energy. The key lies in how to extract the unknown parameters of the PV model. To address this issue, this paper proposes an enhanced neural network algorithm (ENNA). In ENNA, a new transfer operator with three learning strategies based on the defined perturbation operator and elite operator is designed, which makes full use of the obtained population information, including the optimal position of the population, the mean position of the population, and the historical population. To verify the performance of ENNA, ENNA is first used to solve 52 complex benchmark functions. Then, ENNA is employed to extract the unknown parameters of three PV models, i.e., single diode model (SDM), double diode model (DDM), and PV module model (PVM). The optimal root mean square errors obtained by ENNA in SDM, DDM, and PVM are 0.00098602, 0.000982485, and 0.00242507, respectively. ENNA is compared with 10 powerful metaheuristics in terms of numerical comparison, average ranking and convergence performance, and its optimal solutions are compared with the reported optimal solutions of 10 metaheuristic algorithms. The experimental results have demonstrated the excellent performance of ENNA in PV model parameter estimation. The source code of ENNA can be obtained by https://ww2.mathworks.cn/matlabcentral/fileexchange/182977-enna.

## Introduction

At present, traditional energy sources, such as oil, coal, and natural gas, are the main fuels for power generation. However, these traditional energy sources are not only non-renewable but also cause harmful greenhouse gas emissions. As environmental pollution worsens, developing clean energy has become a common goal for many countries. At present, many clean energy sources have been used in real life, such as wind energy, biomass energy, solar energy, geothermal energy, water energy, etc. Compared with other clean energy sources, solar energy has significant advantages:

- Solar energy is not restricted by location and can be obtained anywhere, it will not be exhausted.
- Solar energy is free and safe, has no noise, and is free of pollution.
- Solar power generation does not require complex and expensive infrastructure.
- Ordinary households can also generate electricity through installed solar modules. This electricity can not only be used by the household but can also be sold to earn profits.

Thanks to these advantages, solar energy is considered to be one of the most promising new energy sources. Solar energy can be directly converted into electricity by the photovoltaic (PV) power generation systems. To design, evaluate, and control a PV system correctly, an accurate PV model is needed. The PV power generation system can be simulated, evaluated, and controlled by establishing effective PV models and extracting accurate model parameters. Therefore, parameter extraction of PV models is a key step for PV modeling and system optimization.

In practice, PV models are usually built from equivalent circuit models, and their parameters must be identified from measured data. In the reported literature, the single diode model (SDM) and the double diode model (DDM) are two popular PV models. SDM has five unknown parameters, i.e., the photogenerated current of the PV cell, the current of the diode, the parallel resistance, the series resistance, and the diode ideal factor. DDM has seven unknown parameters, i.e., the photogenerated current of the PV cell, the current of the first diode, the current of the second diode, the parallel resistance, the series resistance, the ideal factor of the first diode, and the ideal factor of the second diode. Since these parameters are coupled in a nonlinear way, the extraction problem is commonly solved as an optimization task. In recent years, many different types of metaheuristic algorithms have been presented to extract the unknown parameters of PV models. For example, the following studies report metaheuristic-based solutions for SDM, DDM, and practical PV modules. Liu et al.^1^ used a multi-strategy adaptive guidance differential evolution algorithm (MSAGDE) for the parameter extraction of SDM, DDM, Photowatt-PWP201, STM6-40/36, and STP6-120/36. The key idea of MSAGDE is based on the opposition-based learning and fitness-distance balance. Oliva et al.^2^ employed an improved chaotic whale optimization algorithm (CWOA) to estimate the parameters of DDM and SDM. The main idea of CWOA is to compute and automatically adapt the internal parameters of CWOA by the designed chaotic maps^2^.

Abbassi et al.^3^ proposed an opposition-based learning modified salp swarm algorithm (OLMSSA) to identify the DDM parameters of the electrical equivalent circuit of the PV cell/module. The core idea of OLMSSA is based on the theory of opposition-based learning, random leader salps, and dynamic transition conditions. Yu et al.^4^ presented a self-adaptive teaching-learning-based optimization (SATLBO) to extract the parameters of a basic commercial solar cell (a commercial R.T.C. France silicon solar cell of 57 mm diameter) and a basic PV module (Photowatt-PWP201 with 36 polycrystalline silicon cells). The primary idea of SATLBO is based on the designed adaptive selection mechanism in the learner phase and the elite learning strategy. Jiao et al.^5^ utilized an orthogonally adapted Harris Hawks optimization (OAHHO) to extract the parameters of the RTC France photovoltaic cell and Photowatt-PWP 201 photovoltaic module. The core idea of OAHHO is based on orthogonal learning and general opposition-based learning. Liang et al.^6^ adopted a self-adaptive ensemble-based differential evolution (SEDE) to estimate the parameters of a 57 mm diameter commercial R.T.C. France silicon solar cells and Photowatt-PWP201 with 36 polycrystalline silicon cells. SEDE uses three different mutation strategies with different characteristics. Abdel-Basset et al.^7^ showed an improved marine predators algorithm (IMPA) to get the parameters of Photowatt-PWP201, STM6-40/36, and STP6-120/36. The core idea of IMPA is based on the population improvement strategy. Long et al.^8^ solved the parameters estimation of SDM, DDM, and PV module by a novel hybrid seagull optimization algorithm (HSOA). The mainidea of HSOA is based on the personal historical best information and the nonlinear escaping energy factor.

Lucas et al.^9^ handled the parameter extraction of RTC France silicon cell, STM6-40 monocrystalline silicon module, and PVM 752 GaAs thin film cell by an enhanced Lévy flight bat algorithm (ELFBA). The core idea of ELFBA is based on the specific mathematical expression to enhance the diversification of new solutions and Lévy flight to perform an effective local search^9^. Gude and Kartick^10^ displayed an improved cuckoo search optimization algorithm (ICSOA) to identify the parameters of SDM, DDM, and PV module model. ICSOA employs an adaptive step size coefficient-based random walk.

According to the experimental results in a number of recent studies^1–3^, metaheuristic algorithms can get promising solutions on the considered parameter extraction of PV models. To explain why these methods are widely used, we summarize their main characteristics below. The characteristics of metaheuristic algorithms can be summarized as follows:

- The search rules defined by metaheuristics are inspired by natural phenomena and are a further simplification of complex natural phenomena, which not only consider the local search but also take into account the global search. Note that these search rules tend to have very simple structures, are very easy to implement, and have low computational complexity.
- Metaheuristic algorithms have a strong ability to escape from local optimal solutions. In the metaheuristic algorithm, individuals cooperate with each other and share the obtained optimal information, and can continuously approach the global optimal solution under defined search rules.
- The introduction of random numbers, such as random numbers uniformly distributed between 0 and 1, random numbers obeying the standard normal distribution, and random numbers obeying the Lévy distribution, can weaken the connection between the random initial solution and the obtained optimal solution.

Although metaheuristics are effective, PV parameter extraction is still challenging for highly nonlinear and multimodal cases. Therefore, researchers also try to design new optimizers by using learning-inspired mechanisms. In the past decade, artificial neural networks (ANNs) have made great progress and have been successfully applied in many engineering fields^11–16^. Shiri et al.^17^ used a neural network-based opportunistic control algorithm to optimize the remote unmanned aerial vehicle online path planning. Qi et al.^18^ investigated the multi-sensor guided hand gesture recognition for a teleoperated robot by a recurrent neural network. Yu et al.^19^ employed a convolutional neural network for medical image analysis, including state-of-the-art comparisons, improvement, and perspectives. Pang et al.^20^ adopted a recurrent neural network to predict the solar radiation. Liu et al.^21^ conducted a survey and performance evaluation of deep neural networks for small object detection. Ding et al.^22^ solved radar-based human activity recognition by a hybrid neural network model with multidomain fusion.

Zhou et al.^23^ designed a partly interpretable convolutional neural network for fault diagnosis of gas turbines. Sultana et al.^24^ presented a deep convolutional neural network for the evolution of image segmentation. Yau et al.^25^ utilized an artificial network model for milling wear prediction. In addition, some scholars have used a combination of artificial neural network technology and metaheuristic algorithms to solve engineering problems, such as the combination of particle swarm optimization and artificial neural networks approach for energy management^26^, the combination of differential evolution algorithm and convolutional neural network for emotional analysis of music data^27^, the combination of cuckoo search and wavelet neural network for midterm building energy forecast^28^, the combination of whale optimization algorithm and convolutional neural network for the heat load prediction model of district heating system^29^, the combination of particle swarm optimization and convolutional neural network for short-term electric load forecasting^30^, the combination of neural network and binary bat algorithm for feature selection^31^, the combination of neural networks and differential evolution algorithm for modeling of oxygen mass transfer in the presence of oxygen-vectors^32^, the combination of whale optimization algorithm and artificial neural network for smart grid cyber intrusion detection^33^.

**Fig. 1:**
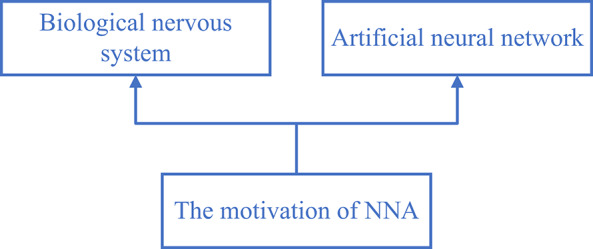
The motivation of NNA.

These studies indicate that neural network concepts can be used not only for prediction, but also as inspirations for designing optimization strategies. As shown in Fig. 1, the neural network algorithm (NNA), inspired by ANNs and the biological nervous system, is a newly proposed metaheuristic algorithm^34^, whose structure is based on feedback ANNs. Specifically, the convergence of NNA has been proven by the authors of NNA^34^. Thanks to the unique structure of feedback ANNs, NNA has demonstrated excellent global search ability. However, when solving complex optimization problems with highly nonlinear objective functions, NNA is prone to getting stuck in local optima. Motivated by the characteristics of NNA, this paper proposes an improved version of NNA for parameter identification of PV models. The contribution of this paper is as follows.

1. This paper proposes an enhanced neural network algorithm (ENNA). The improved strategies introduced to the NNA are to enhance the ability of NNA to escape from the local optima and improve the balance between local search and global search.
2. In ENNA, the improved strategies include the following three aspects. Firstly, a perturbation operator based on random numbers following a standard normal distribution and historical population information is defined. Secondly, an elite operator based on population average position, population optimal position, and random crossover matrix is defined. Lastly, a search mechanism based on defined perturbation operators and elite operators is designed, which uses three search strategies to balance local and global search.
3. ENNA is employed to solve 52 numerical functions with four types and three typical parameter extraction problems from SDM, DDM, and PV module. In addition, the performance comparison between ENNA and 10 powerful metaheuristics is made.

These contributions are important for PV engineering applications. Accurate parameter estimation is essential for reliable PV modeling and performance prediction, because wrong parameters will lead to wrong fitted I–V curves and can also affect key metrics such as the maximum power point (MPP) and efficiency. Also, the objective function for parameter extraction is highly nonlinear and often multimodal, so some existing metaheuristics may converge early or show different results in different runs. For this reason, a more robust and repeatable optimizer is needed for PV parameter extraction.

The rest of this paper is organized as follows. Section “Related work” presents the related work. Section “ENNA” describes the structure and implementation of the proposed ENNA. Experimental results and discussion on the numerical experiment are shown in Section “Numerical experiment”. Experimental results and discussion on parameter extraction of PV models are shown in Section “Parameter extraction of PV models”. The conclusion is made in Section “Conclusion”.

## Related work

### NNA

NNA has a simple structure and is easy to implement, which consists of four parts, i.e., generate trial population, update weight matrix, bias operator, and transfer operator, which will be introduced in the following subsections.

#### Generate trial population

Let ${{\boldsymbol{X}}^{t}}=\left\{ \boldsymbol{x}_{1}^{t},\boldsymbol{x}_{2}^{t},\ldots ,\boldsymbol{x}_{N}^{t} \right\}$ denote a population consisting of *N* individuals, where $\boldsymbol{x}_{i}^{t} (i\in [1,N])$ is the position of individual *i*. Specifically, $\boldsymbol{x}_{i}^{t} (i\in [1,N])$ can be written by $\boldsymbol{x}_{i}^{t}=[x_{i,1}^{t},x_{i,2}^{t},\cdots ,x_{i,D}^{t}]$, where *D* is the number of variables in the solved problem. In NNA, individual *i* has its weight vector $\boldsymbol{w}_{i}^{t}=[w_{i,1}^{t},w_{i,2}^{t},\cdots ,w_{i,N}^{t}]$. All weight vectors form a weight matrix ${{\boldsymbol{W}}^{t}}=\left\{ \boldsymbol{w}_{1}^{t},\boldsymbol{w}_{2}^{t},\ldots ,\boldsymbol{w}_{N}^{t} \right\}$. Thus, the trial population can be generated by^34^:

1 $$\begin{aligned} \boldsymbol{x}_{\text {temp, }j}^{t}=\sum \limits _{i=1}^{N}{w_{i,j}^{t}\times }\boldsymbol{x}_{i}^{t},\,i\in \,\!\![\!\!\text { 1,}N]\,j\in \,\!\![\!\!\text { 1,}N], \end{aligned}$$

2 $$\begin{aligned} \boldsymbol{x}_{\text {trial}, i}^{t}=\boldsymbol{x}_{ i}^{t}+\boldsymbol{x}_{\text {temp,} i}^{t},\,i\in \,\!\![\!\!\text { 1,}N], \end{aligned}$$

where $\boldsymbol{x}_{\text {trial}, i}^{t}$ is the trial individual of individual *i*. All trail individuals form the trial population that can be described by $\boldsymbol{X}_{\text {trial}}^{t}=\left\{ \boldsymbol{x}_{\text {trial}, 1}^{t},\boldsymbol{x}_{\text {trial}, 2}^{t},\ldots ,\boldsymbol{x}_{\text {trial}, N}^{t} \right\}$. In addition, $\boldsymbol{w}_{i}^{t}$ should meet:

3 $$\begin{aligned} \sum \limits _{j=1}^{N}{w_{i,j}^{t}=1,\text { 0}<w_{i,j}^{t}<1,\,i\in \,\!\![\!\!\text { 1,}N],j\in \,\!\![\!\!\text { 1,}N]}. \end{aligned}$$

#### Update weight matrix

${\boldsymbol{W}}^{t}$ is updated by^34^:

4 $$\begin{aligned} \boldsymbol{w}_{i}^{t}=\left| \boldsymbol{w}_{i}^{t}+2\cdot \phi _1 \cdot \left( \boldsymbol{w}_{\text {opt}}^{t}-\boldsymbol{w}_{i}^{t} \right) \right| ,\,i\in \,\!\![\!\!\text { 1,}N], \end{aligned}$$

where $\phi$ is a random number between 0 and 1 and $\boldsymbol{w}_{\text {opt}}^{t}$ is the weight vector corresponding to the obtained best individual. Specifically, if the obtained best individual $\boldsymbol{x}_{\text {opt}}^{t}$ is $\boldsymbol{x}_{j}^{t}\left( j\in \left[ 1,N \right] \right)$, $\boldsymbol{w}_{\text {opt}}^{t}$ is $\boldsymbol{w}_{j}^{t}$.

#### Bias operator

The bias operator in NNA is adjusted by modification factor ${{\beta }^{t}}$, which can be computed by^34^:

5 $$\begin{aligned} {{\beta }^{t+1}}=0.99\cdot {{\beta }^{t}}. \end{aligned}$$

Here, it should be pointed out that ${{\beta }^{0}}$ is initialized by ${{\beta }^{0}}=1$. If individual *i* is selected to perform the bias operator, the process of the bias operator can be described as:

- $\left\lceil \llceil {{\beta }^{t}}\cdot D \right\rceil \rrceil$ variables of $\boldsymbol{x}_{\text {trial}, i}^{t}$ are replaced randomly with the generated randomly variables that meet the upper and lower limits of the variables.
- $\left\lceil \llceil {{\beta }^{t}}\cdot N \right\rceil \rrceil$ weight vectors of ${{\boldsymbol{W}}^{t}}$ are replaced randomly with the generated randomly variables that meet the numbers following a uniform distribution between 0 and 1.

#### Transfer operator

The transfer operator can be represented by^34^:

6 $$\begin{aligned} \boldsymbol{x}_{i}^{t+1}=\boldsymbol{x}_{\text {trial}, i}^{t}+2\cdot \phi _2 \cdot \left( \boldsymbol{x}_{\text {opt}}^{t}-\boldsymbol{x}_{\text {trial}, i}^{t} \right) ,\text {}i \in [1,N], \end{aligned}$$

where $\phi _2$ is a random number with a uniform distribution between 0 and 1. In addition, like other metaheuristic algorithms, ${{\boldsymbol{X}}^{t}}$ in NNA is also randomly initialized^34^:

7 $$\begin{aligned} \boldsymbol{x}_{i}^t = \boldsymbol{l} + \left( {\boldsymbol{u} - \boldsymbol{l}} \right) \cdot \phi _3 ,\mathrm{ } i \in [1,N], \end{aligned}$$

where $\phi _3$ is a random number between 0 and 1, $\boldsymbol{l}$ is the lower limit of the variables in the solved problem, and $\boldsymbol{u}$ is the upper limit of the variables om the solved problem. In addition, the pseudocode of NNA has been presented in Algorithm 1. Algorithm 1 can be stated as follows. The first line represents the parameters for initializing NNA, including weight vectors, population, and modification factor. The second line represents finding the optimal individual and its corresponding weight vector by evaluating individuals in the population. The third to fifteenth lines are the main loop of NNA. The fourth line is to generate the experimental matrix in preparation for performing bias and transfer operations. The seventh and eighth lines are for performing bias and transfer operations, respectively. The twelfth and thirteenth lines are for updating the modification factor and iteration times, respectively. The fourteenth line represents finding the optimal individual and its corresponding weight vector by evaluating individuals in the population. The sixteenth line is outputting the optimal solution.

**Algorithm 1:**
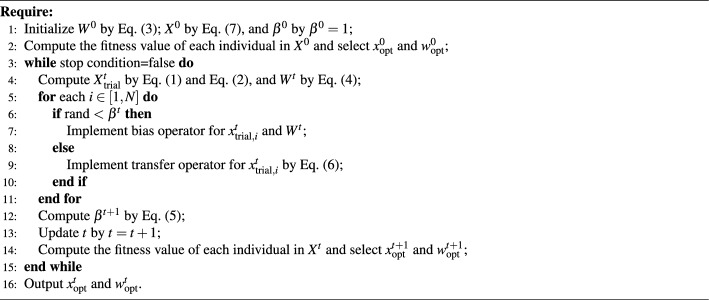
The pseudocode of NNA.

**Fig. 2:**
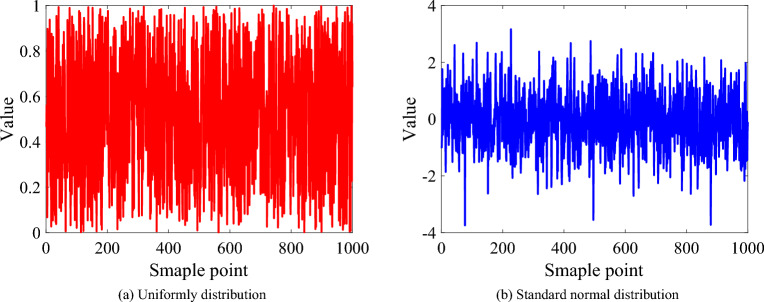
Comparison between uniform distribution between 0 and 1 and standard normal distribution.

### Random numbers obeying standard normal distribution

As mentioned in Section, random numbers are one of the main differences between metaheuristic algorithms and traditional numerical optimization algorithms. The random numbers in the metaheuristic algorithm can not only weaken the connection between the initialization population and the obtained optimal solution but also enhance the global search capability of the algorithm. From Fig. 2(a)and Fig. 2(b), compared with random numbers that obey a uniform distribution between 0 and 1, random numbers that obey a standard normal distribution have a larger range of variation and stronger randomness. In other words, random numbers obeying the standard normal distribution can better highlight the advantages of metaheuristics to a certain extent.

In fact, random numbers obeying a standard normal distribution a are more common in metaheuristic algorithms. In the backtracking search algorithm, authors introduce the random numbers with standard normal distribution to the designed mutation operator^35^. In the generalized normal distribution optimization algorithm, the authors apply the random numbers with standard normal distribution to the designed global exploration stage^36^. In the stochastic fractal search algorithm, the authors use random numbers with a standard normal distribution to generate the randomly selected point^37^. In the dandelion optimizer, authors employ a random number with a standard normal distribution to design the rising stage and descending stage^38^. In the mine last algorithm, the authors adopt a random number with a standard normal distribution to define the search rule^39^. In the human mental search algorithm, authors utilize a random number with a standard normal distribution in the process of generating the step size^40^. In the Newton-raphson-based optimizer, the authors design the search rule based on a random number with a standard normal distribution^41^.

### Mean position of the population

For a metaheuristic algorithm, the change of population position during the search process is roughly divided into three stages, which can be described as follows. In the early stage of the search, the location of the individual is randomly generated. Therefore, there is no regularity in the distribution of individual positions in the population. As the search process progresses, most individuals gradually move toward the location of the optimal individual. In the later stage of the search, most individuals conduct local searches around the location of the optimal individual. Thus, the mean position of the population is a powerful indicator for measuring population characteristics. Therefore, many metaheuristic algorithms introduce the average position of the population in the design of search rules. In the generalized normal distribution optimization algorithm proposed by Zhang et al.^36^, the mean position of the population participates in the design of global search rules. In the teaching-learning-based optimization algorithm proposed by Rao et al.^42^, the mean position of the population is the main parameter in the teacher phase.

In the competitive swarm optimizer proposed by Cheng and Jin^43^, the mean position of the population is a very important parameter in the designed disturbance term. In the proposed adaptive differential evolution with enhanced diversity and restart mechanism by Lin and Meng^44^, the mean position of the population is a key parameter in the designed search rule. In the group teaching optimization algorithm proposed by Zhang and Jin^45^, the mean position of the population plays an important role in the teacher phase. In the hybrid quantum-behaved particle swarm optimization algorithm proposed by Yang et al.^46^, the mean position of the population is the core of the search rule. In the generalized normal distribution optimization proposed by Zhang et al.^36^, the mean position of the population is employed to generate the generalized mean position.

## ENNA

### Motivation

The motivation for ENNA is based on the disadvantages of NNA and the characteristics of the PV model parameter extraction problem. The disadvantages of NNA are summarized as follows:

- The local exploration and global search of NNA are severely imbalanced in the later stage of the search. As shown in line 6 of Algorithm 1 and Eq. (5), in the later stage of the search, the value of A becomes very small, and the probability of the bias operator being performed is very low. That is, in the later stage of the search, NNA mainly performs the transfer operator. According to Eq. (6), the transfer operator is guided by the obtained best solution. Obviously, once the best solution obtained falls into a local optimum, the entire population is easily trapped in a local optimum.
- According to the structure of NNA, the search process is mainly completed through a transfer operator, which only has one search strategy. However, as presented in^47^, search strategies with similar behavior may result in the loss of diversity in the given search region.
- NNA does not have a local escape mechanism, and once the population falls into a local optimal solution, its ability to escape from the local optimal solution is weak. When solving complex multimodal optimization problems using NNA, this phenomenon will become more pronounced.

The characteristics of the PV model parameter extraction problem can be described as follows:

- Its objective function is highly nonlinear. The parameter extraction problem is usually converted into an optimization problem. The expression of the objective function is the root mean square error between the experimental data and the benchmark data. Due to the complex circuit conversion relationships involved, the expression of the objective function often has a high degree of nonlinearity.
- Its objective function has multimodal properties^2^. This characteristic means that there are a large number of local optima in the objective function, which puts very high demands on the algorithm’s global search ability and the ability to jump out of local optima.

In summary, in order to solve the parameter estimation problem of the PV model, the designed improved version of NNA should meet the following requirements:

- It needs to balance local exploration and global search.
- It should have multiple transfer search strategies.
- It should have a strong ability to escape local optimal solutions.

Based on the above analysis, this paper proposes ENNA, which will be introduced in the following subsections.

### The defined two operators in ENNA

#### Perturbation operator

The designed perturbation operator is motivated by the random number obeying the standard normal distribution, which can be described as follows:

8a $$\begin{aligned}&\boldsymbol{x}^t_\text {po1}=\boldsymbol{x}^t_{i}-\boldsymbol{x}^t_{a},\text {if}\, {f(\boldsymbol{x}^t_{i})}\le {f(\boldsymbol{x}^t_{a})}, \end{aligned}$$

8b $$\begin{aligned}&\boldsymbol{x}^t_\text {po1}=\boldsymbol{x}^t_{a}-\boldsymbol{x}^t_{i},\text {if}\, {f(\boldsymbol{x}^t_{i})}>{f(\boldsymbol{x}^t_{a})}, \end{aligned}$$

8c $$\begin{aligned}&\boldsymbol{x}^t_\text {po2}=\boldsymbol{x}^t_{b}-\boldsymbol{x}^t_{c},\text {if}\, {f(\boldsymbol{x}^t_{b})}\le {f(\boldsymbol{x}^t_{c})}, \end{aligned}$$

8d $$\begin{aligned}&\boldsymbol{x}^t_\text {po2}=\boldsymbol{x}^t_{c}-\boldsymbol{x}^t_{b},\text {if}\, {f(\boldsymbol{x}^t_{b})}>{f(\boldsymbol{x}^t_{c})}, \end{aligned}$$

8e $$\begin{aligned}&\boldsymbol{x}^t_{\text {por},i}=\lambda _1\left| \zeta _1\right| \boldsymbol{x}^t_\text {po1}+(1-\lambda _1)\left| \zeta _2\right| \boldsymbol{x}^t_\text {po2}, \end{aligned}$$

where *a*, *b*, and *c* are three integers selected randomly between 1 and *N*, which meet $a\ne b\ne c\ne i$; $\boldsymbol{x}^t_\text {po1}$ is a random learning item and $\boldsymbol{x}^t_\text {po2}$ is a variation learning term; $\lambda _1$ is called the balancing factor and a random number between 0 and 1; $\zeta _1$ and $\zeta _2$ are two random numbers obeying the standard normal distribution; $\boldsymbol{x}^t_{\text {por}, i}$ is the perturbation operator of individual *i*. From Eq. (8), the perturbation properties include the following aspects: 1) three individuals (i.e., individual *a*, individual *b*, and individual *c*) are randomly selected, and are of them are different, 2) the balancing factor is introduced and is a random number between 0 and 1, and can dynamically adjust random learning item and variation learning item, and 3) two random numbers obeying the standard normal distribution are involved, and have stronger volatility compared to uniformly distributed random numbers. Thus, the obtained $\boldsymbol{x}^t_{\text {por},i}$ by Eq. (8) is highly disturbing.

#### Elite operator

The designed elite operator $\boldsymbol{x}^t_\text {eo,i}$ is based on the mean position of the population and the obtained best solution, which can be expressed as follows:

9a $$\begin{aligned}&\boldsymbol{x}^t_\text {eo,i}=\boldsymbol{M}^t_\text {m}\boldsymbol{p}^{t}_i+\boldsymbol{x}_{\text {opt}}^{t}(1-\boldsymbol{p}^{t}_i), \end{aligned}$$

9b $$\begin{aligned}&\boldsymbol{M}^t_\text {m}=\frac{1}{N}\sum _{i=1}^N \boldsymbol{x}^t_i, \end{aligned}$$

where $\boldsymbol{M}^t_\text {m}$ is the mean position of the population, and $\boldsymbol{p}_i$ is the crossover factor of individual *i*, which can be denoted by $\boldsymbol{p}_i=[p_{i,1},p_{i,2},\ldots ,p_{i,D}]$. Further, $\boldsymbol{M}^t_\text {m}$ is the mean position of all individuals in the population. $\boldsymbol{p}_i$ can dynamically adjust the proportion of mean position and historical optimal position in the elite operator. The crossover matrix $\boldsymbol{P}^t (\boldsymbol{P}^t=\{\boldsymbol{p}_i,i\in [1,N]\})$ is motivated by^35^. According to^35^, the method of generating the $\boldsymbol{P}^t$ has been presented in Algorithm 2. As presented in Eq. (9), the defined elite operator reflects both the overall characteristic of the population and the optimal characteristic of the population.

**Algorithm 2:**
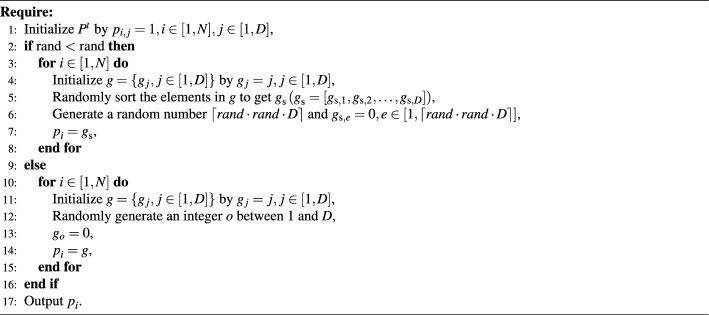
The pseudocode of generating the $\boldsymbol{P}^t$

**Algorithm 3:**
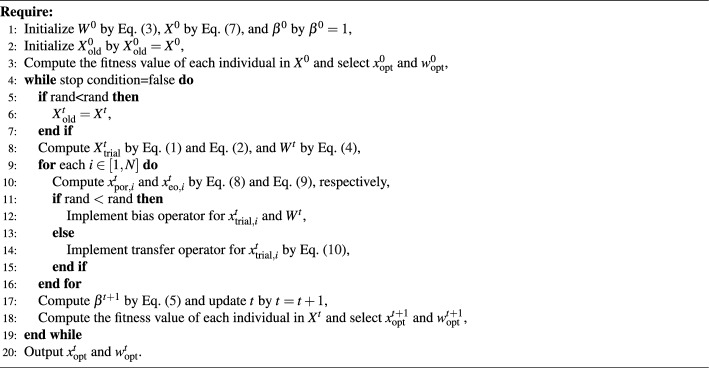
The pseudocode of ENNA.

### The implementation of ENNA

According to the introduced perturbation operator and elite operator, the designed new transfer strategy can be represented as:

10 $$\begin{aligned} \boldsymbol{x}_{i}^{t+1}={\left\{ \begin{array}{ll} \boldsymbol{x}_{\text {trial}, i}^{t}+2\cdot \phi _2 \cdot \left( \boldsymbol{x}_{\text {opt}}^{t}-\boldsymbol{x}_{\text {trial}, i}^{t} \right) +\boldsymbol{x}^t_{\text {por},i},\text {if}\, \lambda _2\le \frac{1}{3},\\ \boldsymbol{x}_{i}^{t}+\phi _3\cdot \zeta _3\cdot \left( \boldsymbol{x}_{i}^{t}-\boldsymbol{x}_{\text {old}, i}^{t} \right) +\boldsymbol{x}^t_{\text {por},i},\text {if}\, \lambda _2\ge \frac{2}{3},\\ \boldsymbol{x}^t_\text {eo,i}+\boldsymbol{x}^t_{\text {por},i},\text {if}\, \frac{1}{3}<\lambda _2<\frac{2}{3}, \end{array}\right. } \end{aligned}$$

where $\phi _3$ and $\lambda _2$ are two random numbers between 0 and 1, $\zeta _3$ are a random number obeying the standard normal distribution, and $\boldsymbol{x}_{\text {old}, i}^{t}$ is the individual *i* in the historical population $\boldsymbol{X}^t_\text {old}=\{\boldsymbol{x}_{\text {old}, i}^{t}, i=1,2,\ldots , N\}$. From Eq. (10), case 1 (i.e., $\lambda _2\le \frac{1}{3}$ is met) means the superposition of the original transfer operator and $\boldsymbol{x}^t_{\text {por}, i}$, which has a stronger ability to escape from the local optimal solutions compared with the original transfer operator; case 2 (i.e., $\lambda _2\ge \frac{2}{3}$ is met) considers the impact of the historical population on the current population, and $\zeta _3$ and $\boldsymbol{x}^t_{\text {por}, i}$ are used to improve the ability of individual *i* to perform the local search; case 3 (i.e., $\frac{1}{3}<\lambda _2<\frac{2}{3}$ is met) is the superposition of $\boldsymbol{x}^t_\text {eo, i}$ and $\boldsymbol{x}^t_{\text {por}, i}$, which is designed to improve the ability of the population to search the global optimal solution. In addition, NNA only has one transfer strategy, while ENNA has three transfer strategies, which means that ENNA is significantly better than NNA in maintaining population diversity. Algorithm 3 shows the pseudocode of ENNA. From Algorithm 3, ENNA not only designs a set of transfer mechanism but also balances the execution probabilities of the transfer operator and bias operator.

The analysis of the computational complexity of ENNA can be described as follows. The initialization phase of ENNA is Line 1 and Line 2 in Algorithm 3, whose computational complexity is $O(3\times N\times D+1)$. Line 3 in Algorithm 3 is to find the global best, whose computational complexity is *O*(*N*). The main loop of ENNA is from Line 4 to Line 19 in Algorithm 3. The computational complexity of Lines 5 to 8 is about $O(0.5\times N\times D+N\times N)$. The computational complexity of Line 10 is $O(5\times N\times D)$. The computational complexity of Lines 11 to 15 is about $O(0.5\times N\times D+(N+1)D+N\times N)$. The computational complexity of Lines 17 to 18 is $O(N+1)$.

**Fig. 3:**
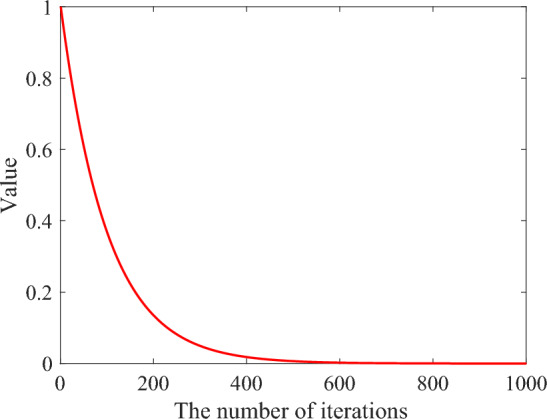
The value of $\beta ^t$ changes with the number of iterations.

Compared with line 06 in Algorithm 2 and line 11 in Algorithm 3, the execution probabilities of ENNA’s bias operator and transfer operator are the same during the search process, which is conducive to giving full play to the advantages of the bias operator and transfer operator. However, the probability of executing the bias operator of NNA is getting smaller and smaller, and the probability of executing the transfer operator is getting larger and larger, as shown in Fig. 3. This is not conducive to the full display of the advantages of the bias operator and increases the probability of the population falling into a locally optimal solution. Thus, ENNA can show better global search ability than NNA. In case 2 (i.e., $\lambda _2\ge \frac{2}{3}$ is met) of Eq. (10), the historical population is introduced, which is motivated by^35^. In^35^, the proposed backtracking search algorithm possesses a memory in which it stores a population from a randomly chosen historical generation for use in generating the search-direction matrix, which helps to enhance the global search ability of the backtracking search algorithm.

**Table 1:** The summary of the employed test suites for comparing the performance between NNA and the other 12 powerful metaheuristic algorithms. “AFs“ means all functions, UFs means unimodal functions, SMFs means simple multimodal functions, HFs means hybrid functions, and CFs means composition functions.

Test suite	AFs	UFs	SMFs	HFs	CFs
CEC 2014^48^	$\text {F}_\text {a1}-\text {F}_\text {a30}$	$\text {F}_\text {a1}-\text {F}_\text {a3}$	$\text {F}_\text {a4}-\text {F}_\text {a16}$	$\text {F}_\text {a17}-\text {F}_\text {a22}$	$\text {F}_\text {a23}-\text {F}_\text {a30}$
CEC 2020^49^	$\text {F}_\text {b1}-\text {F}_\text {b10}$	$\text {F}_\text {b1}$	$\text {F}_\text {b2}-\text {F}_\text {b4}$	$\text {F}_\text {b5}-\text {F}_\text {b7}$	$\text {F}_\text {b8}-\text {F}_\text {b10}$
CEC 2022^50^	$\text {F}_\text {c1}-\text {F}_\text {c12}$	$\text {F}_\text {c1}$	$\text {F}_\text {c2}-\text {F}_\text {c5}$	$\text {F}_\text {c6}-\text {F}_\text {c8}$	$\text {F}_\text {c9}-\text {F}_\text {c12}$

**Table 2:** The experimental results of NNA and ENNA on 30 benchmark functions from CEC 2014 test suite.

No.	Indicator	EO	TSA	SOA	NNA	DE	MVO	WOA	BSA	CLNNA	MLNNA	ENNA
$\text {F}_\text {a1}$	MEAN	7.0E+5	3.6E+8	6.6E+7	9.0E+6	1.2E+6	4.1E+6	5.3E+7	1.2E+6	5.3E+6	1.3E+6	1.6E+5
	STD	4.0E+5	2.4E+8	4.6E+7	6.2E+6	9.7E+5	1.8E+6	2.2E+7	1.4E+6	2.4E+6	8.5E+5	8.8E+4
$\text {F}_\text {a2}$	MEAN	2.3E+2	3.1E+10	8.9E+9	1.5E+4	9.0E+7	3.6E+4	4.6E+7	2.0E+2	1.6E+4	9.7E+3	2.0E+2
	STD	7.2E+1	8.5E+9	4.0E+9	1.1E+4	2.8E+8	1.2E+4	5.0E+7	2.8E+0	8.6E+3	1.1E+4	2.7E+0
$\text {F}_\text {a3}$	MEAN	5.2E+2	4.7E+4	3.6E+4	5.0E+3	2.4E+3	7.1E+2	4.6E+4	6.1E+2	3.6E+3	2.0E+3	3.0E+2
	STD	3.4E+2	1.1E+4	9.5E+3	3.3E+3	2.2E+3	1.4E+2	3.3E+4	5.4E+2	2.7E+3	2.1E+3	2.7E+0
$\text {F}_\text {a4}$	MEAN	4.8E+2	3.0E+3	9.5E+2	5.3E+2	4.8E+2	5.0E+2	6.4E+2	4.9E+2	5.0E+2	4.9E+2	4.4E+2
	STD	3.2E+1	1.3E+3	2.6E+2	4.7E+1	3.3E+1	3.0E+1	6.8E+1	3.7E+1	3.5E+1	3.0E+1	4.5E+1
$\text {F}_\text {a5}$	MEAN	5.2E+2	5.2E+2	5.2E+2	5.2E+2	5.2E+2	5.2E+2	5.2E+2	5.2E+2	5.2E+2	5.2E+2	5.2E+2
	STD	1.4E-1	4.7E-2	7.2E-2	2.0E-1	1.6E-1	4.8E-2	1.4E-1	1.4E-1	5.7E-2	1.6E-1	1.6E-1
$\text {F}_\text {a6}$	MEAN	6.1E+2	6.3E+2	6.3E+2	6.2E+2	6.1E+2	6.1E+2	6.4E+2	6.2E+2	6.2E+2	6.2E+2	6.2E+2
	STD	3.1E+0	2.9E+0	2.8E+0	2.9E+0	3.4E+0	3.4E+0	3.1E+0	7.6E+0	2.7E+0	3.0E+0	3.0E+0
$\text {F}_\text {a7}$	MEAN	7.0E+2	9.7E+2	7.8E+2	7.0E+2	7.0E+2	7.0E+2	7.0E+2	7.0E+2	7.0E+2	7.0E+2	7.0E+2
	STD	1.2E-2	8.0E+1	3.4E+1	6.8E-2	1.6E+0	3.3E-2	4.0E-1	2.1E-2	6.6E-2	7.7E-2	4.0E-2
$\text {F}_\text {a8}$	MEAN	8.6E+2	1.0E+3	9.6E+2	8.6E+2	8.3E+2	8.7E+2	9.8E+2	8.3E+2	8.2E+2	8.8E+2	8.0E+2
	STD	1.7E+1	4.0E+1	3.1E+1	1.7E+1	6.7E+0	1.8E+1	3.8E+1	1.0E+1	5.0E+0	2.0E+1	6.1E-8
$\text {F}_\text {a9}$	MEAN	9.9E+2	1.2E+3	1.1E+3	1.1E+3	9.4E+2	1.0E+3	1.1E+3	9.8E+2	1.0E+3	1.0E+3	1.0E+3
	STD	1.9E+1	5.4E+1	3.1E+1	3.5E+1	1.3E+1	2.4E+1	3.6E+1	1.9E+1	2.8E+1	3.5E+1	2.3E+1
$\text {F}_\text {a10}$	MEAN	2.7E+3	5.7E+3	5.3E+3	2.4E+3	1.9E+3	3.7E+3	5.2E+3	2.5E+3	1.2E+3	2.9E+3	1.0E+3
	STD	4.8E+2	6.8E+2	6.8E+2	4.9E+2	4.5E+2	5.0E+2	7.5E+2	5.8E+2	1.2E+2	5.5E+2	2.5E+1
$\text {F}_\text {a11}$	MEAN	4.5E+3	6.7E+3	5.5E+3	4.9E+3	4.8E+3	4.2E+3	6.4E+3	5.9E+3	4.2E+3	4.9E+3	3.4E+3
	STD	7.3E+2	6.4E+2	7.6E+2	6.7E+2	1.7E+3	6.3E+2	8.5E+2	1.1E+3	6.1E+2	7.1E+2	6.0E+2
$\text {F}_\text {a12}$	MEAN	1.2E+3	1.2E+3	1.2E+3	1.2E+3	1.2E+3	1.2E+3	1.2E+3	1.2E+3	1.2E+3	1.2E+3	1.2E+3
	STD	4.1E-1	3.4E-1	4.9E-1	2.1E-1	1.0E+0	1.5E-1	4.8E-1	3.3E-1	1.7E-1	2.3E-1	1.6E-1
$\text {F}_\text {a13}$	MEAN	1.3E+3	1.3E+3	1.3E+3	1.3E+3	1.3E+3	1.3E+3	1.3E+3	1.3E+3	1.3E+3	1.3E+3	1.3E+3
	STD	8.5E-2	7.0E-1	8.9E-1	1.3E-1	7.4E-2	1.0E-1	1.2E-1	6.6E-2	8.5E-2	1.2E-1	1.1E-1
$\text {F}_\text {a14}$	MEAN	1.4E+3	1.5E+3	1.4E+3	1.4E+3	1.4E+3	1.4E+3	1.4E+3	1.4E+3	1.4E+3	1.4E+3	1.4E+3
	STD	8.2E-2	3.3E+1	1.0E+1	3.4E-1	1.4E-1	3.3E-1	1.0E-1	1.1E-1	4.3E-2	2.8E-1	2.2E-1
$\text {F}_\text {a15}$	MEAN	1.5E+3	2.8E+4	3.5E+3	1.5E+3	1.5E+3	1.5E+3	1.6E+3	1.5E+3	1.5E+3	1.5E+3	1.5E+3
	STD	2.7E+0	3.0E+4	2.3E+3	1.0E+1	8.6E+0	2.5E+0	2.9E+1	4.5E+0	6.5E+0	6.4E+0	7.4E+0
$\text {F}_\text {a16}$	MEAN	1.6E+3	1.6E+3	1.6E+3	1.6E+3	1.6E+3	1.6E+3	1.6E+3	1.6E+3	1.6E+3	1.6E+3	1.6E+3
	STD	7.5E-1	4.4E-1	4.8E-1	4.7E-1	9.3E-1	5.7E-1	5.6E-1	5.5E-1	4.9E-1	5.0E-1	7.8E-1
$\text {F}_\text {a17}$	MEAN	2.4E+5	1.4E+7	1.6E+6	7.5E+5	1.0E+5	2.4E+5	6.5E+6	8.6E+4	3.8E+5	2.6E+5	1.6E+4
	STD	1.4E+5	2.3E+7	1.4E+6	7.0E+5	6.7E+4	1.5E+5	4.4E+6	3.2E+5	2.6E+5	1.5E+5	1.6E+4
$\text {F}_\text {a18}$	MEAN	4.8E+3	7.6E+8	3.4E+7	6.6E+3	6.7E+3	9.5E+3	2.1E+4	2.9E+7	3.4E+3	9.2E+3	3.0E+3
	STD	3.0E+3	1.5E+9	2.2E+7	6.3E+3	5.4E+3	7.7E+3	6.6E+4	1.7E+8	1.8E+3	7.3E+3	2.2E+3
$\text {F}_\text {a19}$	MEAN	1.9E+3	2.1E+3	2.0E+3	1.9E+3	1.9E+3	1.9E+3	2.0E+3	1.9E+3	1.9E+3	1.9E+3	1.9E+3
	STD	8.8E+0	1.0E+2	2.9E+1	2.9E+1	1.4E+1	1.0E+1	3.7E+1	1.2E+1	1.9E+1	2.2E+1	2.0E+1
$\text {F}_\text {a20}$	MEAN	3.1E+3	5.3E+4	1.9E+4	1.7E+4	6.6E+3	2.4E+3	3.6E+4	4.0E+3	6.3E+3	7.2E+3	2.3E+3
	STD	1.1E+3	4.3E+4	8.8E+3	7.0E+3	4.9E+3	1.0E+2	2.1E+4	3.8E+3	2.7E+3	3.4E+3	7.1E+2
$\text {F}_\text {a21}$	MEAN	1.1E+5	6.2E+6	6.8E+5	2.9E+5	3.5E+4	8.1E+4	2.4E+6	1.8E+4	1.6E+5	1.2E+5	1.2E+4
	STD	9.0E+4	8.2E+6	6.6E+5	3.0E+5	3.3E+4	6.1E+4	2.6E+6	1.3E+4	1.2E+5	1.3E+5	1.1E+4
$\text {F}_\text {a22}$	MEAN	2.6E+3	3.9E+3	2.7E+3	2.8E+3	2.4E+3	2.6E+3	3.0E+3	2.5E+3	2.7E+3	2.7E+3	2.7E+3
	STD	2.1E+2	3.2E+3	2.1E+2	1.8E+2	1.9E+2	1.7E+2	2.5E+2	1.2E+2	2.2E+2	2.1E+2	1.8E+2
$\text {F}_\text {a23}$	MEAN	2.6E+3	2.7E+3	2.7E+3	2.6E+3	2.6E+3	2.6E+3	2.6E+3	2.6E+3	2.6E+3	2.6E+3	2.6E+3
	STD	7.6E-12	9.4E+1	1.2E+1	6.7E-1	3.8E+0	6.8E-1	3.0E+1	1.1E-9	1.6E+1	6.8E-7	1.2E-11
$\text {F}_\text {a24}$	MEAN	2.6E+3	2.6E+3	2.6E+3	2.6E+3	2.6E+3	2.6E+3	2.6E+3	2.6E+3	2.6E+3	2.6E+3	2.6E+3
	STD	1.1E-3	8.5E+0	1.1E-3	1.6E+1	7.7E+0	1.3E+1	4.1E+0	4.8E+0	3.5E-1	1.3E+1	1.1E+1
$\text {F}_\text {a25}$	MEAN	2.7E+3	2.7E+3	2.7E+3	2.7E+3	2.7E+3	2.7E+3	2.7E+3	2.7E+3	2.7E+3	2.7E+3	2.7E+3
	STD	4.6E+0	8.7E+0	7.8E+0	7.2E+0	1.2E+0	1.6E+0	1.6E+1	4.4E+0	7.0E-5	4.4E+0	7.0E+0
$\text {F}_\text {a26}$	MEAN	2.7E+3	2.8E+3	2.7E+3	2.7E+3	2.7E+3	2.7E+3	2.7E+3	2.7E+3	2.7E+3	2.7E+3	2.7E+3
	STD	4.8E+1	5.7E+1	3.8E-1	1.4E-1	2.0E+1	6.3E+1	4.7E+1	1.5E+0	2.0E+1	1.2E-1	1.3E-1
$\text {F}_\text {a27}$	MEAN	3.2E+3	3.8E+3	3.7E+3	3.3E+3	3.2E+3	3.3E+3	3.8E+3	3.7E+3	3.4E+3	3.4E+3	3.5E+3
	STD	9.5E+1	3.0E+2	1.6E+2	2.9E+2	8.6E+1	1.1E+2	3.5E+2	2.8E+2	2.3E+2	2.4E+2	2.1E+2
$\text {F}_\text {a28}$	MEAN	3.8E+3	7.2E+3	4.0E+3	4.2E+3	3.9E+3	3.9E+3	5.2E+3	4.1E+3	4.0E+3	3.9E+3	4.0E+3
	STD	1.6E+2	1.0E+3	2.6E+2	4.4E+2	1.7E+2	2.4E+2	6.0E+2	3.6E+2	3.3E+2	1.7E+2	2.4E+2
$\text {F}_\text {a29}$	MEAN	1.5E+6	5.9E+7	4.3E+6	1.1E+6	2.4E+6	1.6E+6	7.7E+6	8.5E+6	5.1E+5	1.8E+6	3.6E+6
	STD	3.5E+6	4.0E+7	3.9E+6	2.9E+6	4.8E+6	4.1E+6	4.9E+6	2.5E+6	2.0E+6	3.5E+6	4.2E+6
$\text {F}_\text {a30}$	MEAN	8.0E+3	3.6E+5	8.0E+4	1.2E+4	9.1E+3	9.5E+3	1.2E+5	9.7E+4	6.4E+3	7.5E+3	5.5E+3
	STD	6.3E+3	3.8E+5	5.3E+4	5.3E+3	8.8E+3	2.6E+3	9.3E+4	1.2E+5	9.3E+2	1.3E+3	1.1E+3

**Table 3:** The experimental results of NNA and ENNA on 10 benchmark functions from CEC 2020 test suite.

No.	Indicator	EO	TSA	SOA	NNA	DE	MVO	WOA	BSA	CLNNA	MLNNA	ENNA
$\text {F}_\text {b1}$	MEAN	7.0E+5	3.6E+8	6.6E+7	9.0E+6	1.2E+6	4.1E+6	5.3E+7	1.2E+6	5.3E+6	1.3E+6	1.6E+5
	STD	4.0E+5	2.4E+8	4.6E+7	6.2E+6	9.7E+5	1.8E+6	2.2E+7	1.4E+6	2.4E+6	8.5E+5	8.8E+4
$\text {F}_\text {b2}$	MEAN	2.3E+2	3.1E+10	8.9E+9	1.5E+4	9.0E+7	3.6E+4	4.6E+7	2.0E+2	1.6E+4	9.7E+3	2.0E+2
	STD	7.2E+1	8.5E+9	4.0E+9	1.1E+4	2.8E+8	1.2E+4	5.0E+7	2.8E+0	8.6E+3	1.1E+4	2.7E+0
$\text {F}_\text {b3}$	MEAN	5.2E+2	4.7E+4	3.6E+4	5.0E+3	2.4E+3	7.1E+2	4.6E+4	6.1E+2	3.6E+3	2.0E+3	3.0E+2
	STD	3.4E+2	1.1E+4	9.5E+3	3.3E+3	2.2E+3	1.4E+2	3.3E+4	5.4E+2	2.7E+3	2.1E+3	2.7E+0
$\text {F}_\text {b4}$	MEAN	4.8E+2	3.0E+3	9.5E+2	5.3E+2	4.8E+2	5.0E+2	6.4E+2	4.9E+2	5.0E+2	4.9E+2	4.4E+2
	STD	3.2E+1	1.3E+3	2.6E+2	4.7E+1	3.3E+1	3.0E+1	6.8E+1	3.7E+1	3.5E+1	3.0E+1	4.5E+1
$\text {F}_\text {b5}$	MEAN	5.2E+2	5.2E+2	5.2E+2	5.2E+2	5.2E+2	5.2E+2	5.2E+2	5.2E+2	5.2E+2	5.2E+2	5.2E+2
	STD	1.4E-1	4.7E-2	7.2E-2	2.0E-1	1.6E-1	4.8E-2	1.4E-1	1.4E-1	5.7E-2	1.6E-1	1.6E-1
$\text {F}_\text {b6}$	MEAN	6.1E+2	6.3E+2	6.3E+2	6.2E+2	6.1E+2	6.1E+2	6.4E+2	6.2E+2	6.2E+2	6.2E+2	6.2E+2
	STD	3.1E+0	2.9E+0	2.8E+0	2.9E+0	3.4E+0	3.4E+0	3.1E+0	7.6E+0	2.7E+0	3.0E+0	3.0E+0
$\text {F}_\text {b7}$	MEAN	7.0E+2	9.7E+2	7.8E+2	7.0E+2	7.0E+2	7.0E+2	7.0E+2	7.0E+2	7.0E+2	7.0E+2	7.0E+2
	STD	1.2E-2	8.0E+1	3.4E+1	6.8E-2	1.6E+0	3.3E-2	4.0E-1	2.1E-2	6.6E-2	7.7E-2	4.0E-2
$\text {F}_\text {b8}$	MEAN	8.6E+2	1.0E+3	9.6E+2	8.6E+2	8.3E+2	8.7E+2	9.8E+2	8.3E+2	8.2E+2	8.8E+2	8.0E+2
	STD	1.7E+1	4.0E+1	3.1E+1	1.7E+1	6.7E+0	1.8E+1	3.8E+1	1.0E+1	5.0E+0	2.0E+1	6.1E-8
$\text {F}_\text {b9}$	MEAN	9.9E+2	1.2E+3	1.1E+3	1.1E+3	9.4E+2	1.0E+3	1.1E+3	9.8E+2	1.0E+3	1.0E+3	1.0E+3
	STD	1.9E+1	5.4E+1	3.1E+1	3.5E+1	1.3E+1	2.4E+1	3.6E+1	1.9E+1	2.8E+1	3.5E+1	2.3E+1
$\text {F}_\text {b10}$	MEAN	2.7E+3	5.7E+3	5.3E+3	2.4E+3	1.9E+3	3.7E+3	5.2E+3	2.5E+3	1.2E+3	2.9E+3	1.0E+3
	STD	4.8E+2	6.8E+2	6.8E+2	4.9E+2	4.5E+2	5.0E+2	7.5E+2	5.8E+2	1.2E+2	5.5E+2	2.5E+1

**Table 4:** The experimental results of NNA and ENNA on 30 benchmark functions from CEC 2022 test suite.

No.	Indicator	EO	TSA	SOA	NNA	DE	MVO	WOA	BSA	CLNNA	MLNNA	ENNA
$\text {F}_\text {c1}$	MEAN	7.0E+5	3.6E+8	6.6E+7	9.0E+6	1.2E+6	4.1E+6	5.3E+7	1.2E+6	5.3E+6	1.3E+6	1.6E+5
	STD	4.0E+5	2.4E+8	4.6E+7	6.2E+6	9.7E+5	1.8E+6	2.2E+7	1.4E+6	2.4E+6	8.5E+5	8.8E+4
$\text {F}_\text {c2}$	MEAN	2.3E+2	3.1E+10	8.9E+9	1.5E+4	9.0E+7	3.6E+4	4.6E+7	2.0E+2	1.6E+4	9.7E+3	2.0E+2
	STD	7.2E+1	8.5E+9	4.0E+9	1.1E+4	2.8E+8	1.2E+4	5.0E+7	2.8E+0	8.6E+3	1.1E+4	2.7E+0
$\text {F}_\text {c3}$	MEAN	5.2E+2	4.7E+4	3.6E+4	5.0E+3	2.4E+3	7.1E+2	4.6E+4	6.1E+2	3.6E+3	2.0E+3	3.0E+2
	STD	3.4E+2	1.1E+4	9.5E+3	3.3E+3	2.2E+3	1.4E+2	3.3E+4	5.4E+2	2.7E+3	2.1E+3	2.7E+0
$\text {F}_\text {c4}$	MEAN	4.8E+2	3.0E+3	9.5E+2	5.3E+2	4.8E+2	5.0E+2	6.4E+2	4.9E+2	5.0E+2	4.9E+2	4.4E+2
	STD	3.2E+1	1.3E+3	2.6E+2	4.7E+1	3.3E+1	3.0E+1	6.8E+1	3.7E+1	3.5E+1	3.0E+1	4.5E+1
$\text {F}_\text {c5}$	MEAN	5.2E+2	5.2E+2	5.2E+2	5.2E+2	5.2E+2	5.2E+2	5.2E+2	5.2E+2	5.2E+2	5.2E+2	5.2E+2
	STD	1.4E-1	4.7E-2	7.2E-2	2.0E-1	1.6E-1	4.8E-2	1.4E-1	1.4E-1	5.7E-2	1.6E-1	1.6E-1
$\text {F}_\text {c6}$	MEAN	6.1E+2	6.3E+2	6.3E+2	6.2E+2	6.1E+2	6.1E+2	6.4E+2	6.2E+2	6.2E+2	6.2E+2	6.2E+2
	STD	3.1E+0	2.9E+0	2.8E+0	2.9E+0	3.4E+0	3.4E+0	3.1E+0	7.6E+0	2.7E+0	3.0E+0	3.0E+0
$\text {F}_\text {c7}$	MEAN	7.0E+2	9.7E+2	7.8E+2	7.0E+2	7.0E+2	7.0E+2	7.0E+2	7.0E+2	7.0E+2	7.0E+2	7.0E+2
	STD	1.2E-2	8.0E+1	3.4E+1	6.8E-2	1.6E+0	3.3E-2	4.0E-1	2.1E-2	6.6E-2	7.7E-2	4.0E-2
$\text {F}_\text {c8}$	MEAN	8.6E+2	1.0E+3	9.6E+2	8.6E+2	8.3E+2	8.7E+2	9.8E+2	8.3E+2	8.2E+2	8.8E+2	8.0E+2
	STD	1.7E+1	4.0E+1	3.1E+1	1.7E+1	6.7E+0	1.8E+1	3.8E+1	1.0E+1	5.0E+0	2.0E+1	6.1E-8
$\text {F}_\text {c9}$	MEAN	9.9E+2	1.2E+3	1.1E+3	1.1E+3	9.4E+2	1.0E+3	1.1E+3	9.8E+2	1.0E+3	1.0E+3	1.0E+3
	STD	1.9E+1	5.4E+1	3.1E+1	3.5E+1	1.3E+1	2.4E+1	3.6E+1	1.9E+1	2.8E+1	3.5E+1	2.3E+1
$\text {F}_\text {c10}$	MEAN	2.7E+3	5.7E+3	5.3E+3	2.4E+3	1.9E+3	3.7E+3	5.2E+3	2.5E+3	1.2E+3	2.9E+3	1.0E+3
	STD	4.8E+2	6.8E+2	6.8E+2	4.9E+2	4.5E+2	5.0E+2	7.5E+2	5.8E+2	1.2E+2	5.5E+2	2.5E+1
$\text {F}_\text {c11}$	MEAN	4.5E+3	6.7E+3	5.5E+3	4.9E+3	4.8E+3	4.2E+3	6.4E+3	5.9E+3	4.2E+3	4.9E+3	3.4E+3
	STD	7.3E+2	6.4E+2	7.6E+2	6.7E+2	1.7E+3	6.3E+2	8.5E+2	1.1E+3	6.1E+2	7.1E+2	6.0E+2
$\text {F}_\text {c12}$	MEAN	1.2E+3	1.2E+3	1.2E+3	1.2E+3	1.2E+3	1.2E+3	1.2E+3	1.2E+3	1.2E+3	1.2E+3	1.2E+3
	STD	4.1E-1	3.4E-1	4.9E-1	2.1E-1	1.0E+0	1.5E-1	4.8E-1	3.3E-1	1.7E-1	2.3E-1	1.6E-1

**Table 5:** The experimental results between ENNA and the compared algorithms on 30 benchmark functions from CEC 2014 test suite according to Wilcoxon signed ranks test (the significant level is set to 0.05).

No.	ENNA vs.																			
	EO		TSA		SOA		NNA		DE		MVO		WOA		BSA		CLNNA		MLNNA	
	p-value	S	p-value	S	p-value	S	p-value	S	p-value	S	p-value	S	p-value	S	p-value	S	p-value	S	p-value	S
$\text {F}_\text {a1}$	1.7E-09	+	7.6E-10	+	7.6E-10	+	7.6E-10	+	7.6E-10	+	7.6E-10	+	7.6E-10	+	7.6E-10	+	7.6E-10	+	8.0E-10	+
$\text {F}_\text {a2}$	9.1E-10	+	7.6E-10	+	7.6E-10	+	8.0E-10	+	7.6E-10	+	7.6E-10	+	7.6E-10	+	1.3E-01	=	7.6E-10	+	7.6E-10	+
$\text {F}_\text {a3}$	7.6E-10	+	7.6E-10	+	7.6E-10	+	7.6E-10	+	7.6E-10	+	7.6E-10	+	7.6E-10	+	2.0E-09	+	7.6E-10	+	7.6E-10	+
$\text {F}_\text {a4}$	9.2E-06	+	7.6E-10	+	7.6E-10	+	1.3E-08	+	3.9E-06	+	3.5E-08	+	7.6E-10	+	3.4E-06	+	2.0E-07	+	3.9E-08	+
$\text {F}_\text {a5}$	1.3E-04	+	7.6E-10	+	7.6E-10	+	2.4E-06	-	7.6E-10	+	3.8E-09	-	4.3E-03	+	1.0E-09	+	3.4E-09	-	1.8E-07	-
$\text {F}_\text {a6}$	7.6E-10	-	7.6E-10	+	2.4E-09	+	4.1E-08	+	8.0E-10	-	8.5E-10	-	7.6E-10	+	9.0E-03	+	9.0E-03	+	3.7E-02	+
$\text {F}_\text {a7}$	4.9E-05	-	7.6E-10	+	7.6E-10	+	2.4E-04	+	1.3E-06	+	2.5E-08	+	7.6E-10	+	1.6E-03	-	1.1E-06	+	8.9E-01	=
$\text {F}_\text {a8}$	7.6E-10	+	7.6E-10	+	7.6E-10	+	7.6E-10	+	7.6E-10	-	7.6E-10	+	7.6E-10	+	7.6E-10	+	7.6E-10	+	7.6E-10	+
$\text {F}_\text {a9}$	1.0E-01	=	7.6E-10	+	1.1E-09	+	1.1E-08	+	7.6E-10	+	3.6E-01	=	7.6E-10	+	1.3E-04	-	1.5E-07	+	5.0E-04	+
$\text {F}_\text {a10}$	7.6E-10	+	7.6E-10	+	7.6E-10	+	7.6E-10	+	7.6E-10	+	7.6E-10	+	7.6E-10	+	7.6E-10	+	1.2E-09	+	7.6E-10	+
$\text {F}_\text {a11}$	2.8E-08	+	7.6E-10	+	7.6E-10	+	2.0E-09	+	7.0E-06	+	5.1E-06	+	7.6E-10	+	1.9E-09	+	2.3E-07	+	3.8E-09	+
$\text {F}_\text {a12}$	1.1E-09	+	7.6E-10	+	7.6E-10	+	3.0E-09	+	4.0E-09	+	1.6E-01	=	7.6E-10	+	3.8E-09	+	6.3E-05	+	1.5E-08	+
$\text {F}_\text {a13}$	8.5E-10	-	7.6E-10	+	2.5E-09	+	1.1E-02	+	1.6E-09	-	2.2E-05	-	1.1E-02	-	1.2E-09	-	1.6E-06	-	3.9E-01	=
$\text {F}_\text {a14}$	1.4E-05	-	7.6E-10	+	8.0E-10	+	3.1E-05	+	8.6E-01	=	6.9E-02	=	7.2E-04	-	5.2E-04	-	1.8E-07	-	2.9E-06	+
$\text {F}_\text {a15}$	7.6E-10	-	7.6E-10	+	7.6E-10	+	6.6E-05	+	8.0E-06	-	1.3E-09	-	7.6E-10	+	9.6E-07	-	7.2E-01	=	7.9E-01	=
$\text {F}_\text {a16}$	1.9E-09	+	7.6E-10	+	7.6E-10	+	7.6E-10	+	2.2E-09	+	7.6E-10	+	7.6E-10	+	8.0E-10	+	9.1E-10	+	7.6E-10	+
$\text {F}_\text {a17}$	7.6E-10	+	7.6E-10	+	7.6E-10	+	7.6E-10	+	3.4E-09	+	8.0E-10	+	7.6E-10	+	3.1E-06	+	7.6E-10	+	8.0E-10	+
$\text {F}_\text {a18}$	1.9E-04	+	7.6E-10	+	7.6E-10	+	3.1E-05	+	1.8E-05	+	4.8E-07	+	3.8E-09	+	4.2E-06	+	9.0E-03	+	2.0E-07	+
$\text {F}_\text {a19}$	3.7E-06	-	7.6E-10	+	4.8E-09	+	3.5E-05	+	7.0E-08	-	2.8E-01	=	1.3E-07	+	5.9E-07	-	3.4E-03	+	1.0E-03	+
$\text {F}_\text {a20}$	2.6E-06	+	7.6E-10	+	7.6E-10	+	7.6E-10	+	7.6E-09	+	6.3E-05	+	7.6E-10	+	2.0E-07	+	7.6E-10	+	1.7E-09	+
$\text {F}_\text {a21}$	1.8E-09	+	7.6E-10	+	7.6E-10	+	1.4E-09	+	9.6E-06	+	2.2E-09	+	7.6E-10	+	5.7E-03	+	7.6E-10	+	1.2E-09	+
$\text {F}_\text {a22}$	4.1E-03	-	4.3E-07	+	6.6E-01	=	1.2E-02	+	1.3E-07	-	3.3E-02	-	1.0E-06	+	4.5E-08	-	7.4E-01	=	8.3E-01	=
$\text {F}_\text {a23}$	9.4E-05	-	7.6E-10	+	7.6E-10	+	7.6E-10	+	7.6E-10	+	7.6E-10	+	2.0E-07	+	7.0E-09	+	1.4E-08	+	1.4E-09	+
$\text {F}_\text {a24}$	7.6E-10	-	1.3E-09	-	7.6E-10	-	5.7E-01	=	1.9E-06	+	2.7E-02	-	1.1E-09	-	4.1E-03	-	1.1E-09	-	1.8E-01	=
$\text {F}_\text {a25}$	2.5E-07	-	2.1E-09	+	7.4E-01	=	3.0E-03	+	8.2E-08	-	1.6E-05	-	1.3E-02	+	1.3E-01	=	7.6E-10	-	1.4E-01	=
$\text {F}_\text {a26}$	4.4E-01	=	7.6E-10	+	8.0E-10	+	1.7E-04	+	2.9E-01	=	5.2E-02	=	6.5E-01	=	1.5E-02	=	2.9E-05	+	7.4E-03	+
$\text {F}_\text {a27}$	2.1E-08	-	1.1E-05	+	1.3E-05	+	6.7E-04	-	1.4E-08	-	1.3E-07	-	3.1E-05	+	1.3E-02	+	5.6E-04	-	3.5E-05	-
$\text {F}_\text {a28}$	1.8E-05	-	7.6E-10	+	3.4E-01	=	5.5E-03	+	2.9E-03	-	1.6E-02	-	9.1E-10	+	5.3E-02	=	7.7E-01	=	5.9E-03	-
$\text {F}_\text {a29}$	5.5E-01	=	9.1E-10	+	5.9E-02	=	3.6E-01	=	9.2E-01	=	7.2E-01	=	1.3E-07	+	1.1E-06	+	2.4E-02	-	8.4E-01	=
$\text {F}_\text {a30}$	4.2E-05	+	7.6E-10	+	7.6E-10	+	7.6E-10	+	3.1E-05	+	9.1E-10	+	7.6E-10	+	1.3E-09	+	1.2E-04	+	3.9E-08	+
+		15		29		25		26		18		15		26		18		20		20
-		12		1		1		2		9		9		3		8		7		3
=		3		0		4		2		3		6		1		4		3		7

**Table 6:** The experimental results between ENNA and the compared algorithms on 10 benchmark functions from the CEC 2020 test suite according to Wilcoxon signed ranks test (the significance level is set to 0.05).

No.	ENNA vs.																			
	EO		TSA		SOA		NNA		DE		MVO		WOA		BSA		CLNNA		MLNNA	
	p-value	S	p-value	S	p-value	S	p-value	S	p-value	S	p-value	S	p-value	S	p-value	S	p-value	S	p-value	S
$\text {F}_\text {b1}$	8.5E-10	+	7.6E-10	+	7.6E-10	+	7.6E-10	+	7.6E-10	+	7.6E-10	+	7.6E-10	+	7.1E-05	-	7.6E-10	+	7.6E-10	+
$\text {F}_\text {b2}$	1.5E-08	+	7.6E-10	+	7.6E-10	+	8.5E-10	+	1.7E-06	+	9.1E-10	+	7.6E-10	+	2.2E-09	+	3.2E-04	+	7.6E-10	+
$\text {F}_\text {b3}$	3.5E-05	+	7.6E-10	+	7.6E-10	+	8.0E-09	+	1.8E-03	-	1.4E-08	+	7.6E-10	+	2.3E-01	=	9.6E-06	+	7.6E-10	+
$\text {F}_\text {b4}$	1.6E-01	=	7.6E-10	+	7.6E-10	+	1.4E-08	+	5.4E-01	=	2.8E-04	+	7.6E-10	+	1.0E-01	=	9.0E-09	+	1.0E-06	+
$\text {F}_\text {b5}$	7.6E-10	+	7.6E-10	+	7.6E-10	+	7.6E-10	-	3.1E-06	+	8.0E-10	+	7.6E-10	+	1.6E-01	=	7.6E-10	+	7.6E-10	+
$\text {F}_\text {b6}$	7.6E-10	+	7.6E-10	+	7.6E-10	+	7.6E-10	+	7.6E-10	+	7.6E-10	+	7.6E-10	+	7.6E-10	+	1.2E-09	+	7.6E-10	+
$\text {F}_\text {b7}$	7.6E-10	+	7.6E-10	+	7.6E-10	+	7.6E-10	+	8.5E-03	+	7.6E-10	+	7.6E-10	+	3.3E-01	=	7.6E-10	+	7.6E-10	+
$\text {F}_\text {b8}$	4.3E-01	=	7.6E-10	+	7.6E-10	+	7.9E-01	=	1.3E-01	=	9.1E-08	+	7.6E-10	+	3.7E-04	+	4.0E-01	=	3.5E-01	=
$\text {F}_\text {b9}$	2.0E-09	-	7.6E-10	+	7.2E-04	-	4.3E-01	=	8.5E-10	-	7.6E-10	-	1.6E-09	+	1.0E-06	-	1.8E-01	=	2.2E-05	-
$\text {F}_\text {b10}$	5.7E-02	=	9.1E-10	+	1.8E-07	+	1.3E-01	=	3.0E-04	-	5.3E-07	-	6.7E-06	+	1.4E-01	=	5.6E-01	=	2.8E-02	-
+		6		10		9		6		5		8		10		3		7		7
-		1		0		1		1		3		2		0		2		0		2
=		3		0		0		3		2		0		0		5		3		1

**Table 7:** The experimental results between ENNA and the compared algorithms on 12 benchmark functions from the CEC 2022 test suite according to the Wilcoxon signed ranks test (the significance level is set to 0.05).

No.	ENNA vs.																			
	EO		TSA		SOA		NNA		DE		MVO		WOA		BSA		CLNNA		MLNNA	
	p-value	S	p-value	S	p-value	S	p-value	S	p-value	S	p-value	S	p-value	S	p-value	S	p-value	S	p-value	S
$\text {F}_\text {c1}$	7.6E-10	+	7.6E-10	+	7.6E-10	+	7.6E-10	+	8.0E-10	+	7.6E-10	+	7.6E-10	+	7.6E-10	+	7.6E-10	+	7.6E-10	+
$\text {F}_\text {c2}$	2.1E-06	+	7.6E-10	+	7.6E-10	+	7.7E-06	+	2.7E-05	+	1.1E-03	+	7.6E-10	+	1.4E-06	+	9.6E-07	+	1.2E-03	+
$\text {F}_\text {c3}$	4.5E-09	-	7.6E-10	+	7.6E-10	+	7.6E-10	+	7.6E-09	-	7.6E-10	+	7.6E-10	+	4.8E-09	-	7.6E-10	+	7.6E-10	+
$\text {F}_\text {c4}$	5.1E-03	=	7.6E-10	+	8.5E-09	+	2.3E-08	+	2.8E-08	-	1.6E-01	=	7.6E-10	+	1.1E-06	-	3.0E-07	+	6.8E-05	+
$\text {F}_\text {c5}$	7.6E-10	-	7.6E-10	+	1.7E-09	+	1.3E-09	+	7.6E-10	-	9.8E-05	-	7.6E-10	+	3.6E-08	-	1.0E-08	+	2.6E-01	=
$\text {F}_\text {c6}$	4.8E-09	+	7.6E-10	+	7.6E-10	+	8.0E-10	+	2.2E-07	+	1.5E-09	+	8.0E-10	+	9.2E-07	+	7.6E-10	+	1.6E-09	+
$\text {F}_\text {c7}$	2.8E-02	+	7.6E-10	+	3.8E-09	+	2.8E-09	+	7.0E-02	=	3.7E-07	+	7.6E-10	+	1.3E-03	+	1.0E-03	+	5.1E-09	+
$\text {F}_\text {c8}$	4.8E-02	+	4.5E-09	+	2.5E-05	+	1.1E-06	+	1.9E-01	=	3.7E-07	+	5.6E-06	+	1.7E-01	=	2.4E-02	+	4.1E-03	+
$\text {F}_\text {c9}$	1.4E-02	-	7.6E-10	+	7.6E-10	+	7.6E-10	+	1.1E-09	+	7.6E-10	+	7.6E-10	+	3.8E-01	=	7.6E-10	+	7.6E-10	+
$\text {F}_\text {c10}$	1.5E-05	+	7.6E-10	+	2.9E-08	+	6.9E-04	+	6.0E-03	+	2.7E-09	+	8.0E-10	+	1.1E-09	+	7.4E-01	=	3.7E-01	=
$\text {F}_\text {c11}$	1.4E-01	=	7.6E-10	+	7.6E-10	+	3.5E-04	+	6.6E-05	+	4.3E-05	+	3.1E-05	+	7.9E-01	=	4.3E-07	+	1.1E-02	+
$\text {F}_\text {c12}$	1.9E-03	-	8.0E-10	+	8.0E-04	1	3.9E-01	=	3.7E-02	-	1.3E-04	-	7.4E-08	+	1.8E-01	=	7.5E-01	=	1.2E-04	-
+		6		12		11		11		6		9		12		5		10		9
-		4		0		1		0		4		2		0		3		0		1
=		2		0		0		1		2		1		0		4		2		2

## Numerical experiment

This section is to verify the effectiveness of the improved strategies presented in Section by comparing ENNA and 10 powerful metaheuristic algorithms, which are equilibrium optimizer (EO)^51^, tunicate swarm algorithm (TSA)^52^, seagull optimization algorithm (SOA)^53^, NNA^34^, differential evolution (DE)^54^, multi-verse optimizer (MVO)^55^, whale optimization algorithm (WOA)^56^, backtracking search algorithm (BSA)^35^, chaotic neural network algorithm with competitive learning (CLNNA)^57^, and multiple learning neural network algorithm (MLNNA)^58^ on the challenging CEC 2014 test suite^48^, CEC 2017 test suite^59^, CEC 2020 test suite^49^, and CEC 2022 test suite^50^. Note that CLNNA and MLNNA are two recently reported variants of NNA, which can better check the validity of the improved strategies introduced to ENNA. CLNNA is based on the competitive learning and chaos theory to enhance the global search ability of NNA. MLNNA is based on six learning strategies by the designed local elite archive and global elite archive to balance exploration and exploitation of NNA. Unlike CLNNA and MLNNA, ENNA designs bias and transfer operations through defined perturbation and elite operators, thereby enhancing the global optimization performance of NNA. The summary of the four test suites has been described in Table 1. Experiments are simulated using MATLAB software installed on the Windows 11 operating system. The computing system is a core i7 system with 2.3GHz CPU and 16GB memory.

### Comparison on CEC 2014 test suite

This section is to compare the performance between NNA and ENNA on 30 benchmark functions with four types from CEC 2014 test suite^48^, which include three unimodal functions (i.e., $\text {F}_\text {a1}$, $\text {F}_\text {a2}$, and $\text {F}_\text {a3}$), 13 simple multimodal functions (i.e., $\text {F}_\text {a4}$, $\text {F}_\text {a5}$, $\text {F}_\text {a6}$, $\text {F}_\text {a7}$, $\text {F}_\text {a8}$, $\text {F}_\text {a9}$, $\text {F}_\text {a10}$, $\text {F}_\text {a11}$, $\text {F}_\text {a12}$, $\text {F}_\text {a13}$, $\text {F}_\text {a14}$, $\text {F}_\text {a15}$, and $\text {F}_\text {a16}$), six hybrid functions (i.e., $\text {F}_\text {a17}$, $\text {F}_\text {a18}$, $\text {F}_\text {a19}$, $\text {F}_\text {a20}$, $\text {F}_\text {a21}$, and $\text {F}_\text {a22}$), and eight composition functions ($\text {F}_\text {a23}$, $\text {F}_\text {a24}$, $\text {F}_\text {a25}$, $\text {F}_\text {a26}$, $\text {F}_\text {a27}$, $\text {F}_\text {a28}$, $\text {F}_\text {a29}$, and $\text {F}_\text {a30}$). In this experiment, the dimension of each function is set to 30; the population size of each algorithm is set to 30; the maximum number of iterations is set to 5,000; the number of independent repeated experiments is set to 50. The other control parameters of the compared algorithms are extracted from the corresponding original references.

Table 2 shows the obtained experimental results of NNA and ENNA in 30 benchmark functions. Specifically, in Table 2, MEAN and STD represent the mean value and standard deviation, respectively. Looking at Table 2, in terms of MEAN, ENNA can get the best MEAN in 11 test functions, which are $\text {F}_\text {a1}$, $\text {F}_\text {a3}$, $\text {F}_\text {a4}$, $\text {F}_\text {a8}$, $\text {F}_\text {a10}$, $\text {F}_\text {a11}$, $\text {F}_\text {a17}$, $\text {F}_\text {a18}$, $\text {F}_\text {a20}$, $\text {F}_\text {a21}$, and $\text {F}_\text {a30}$. In addition, ENNA and BSA can share the best MEAN in $\text {F}_\text {a5}$, ENNA can share the best MEAN with EO, NNA, DE, MVO, WOA, BSA, MLNNA, and CLNNA in $\text {F}_\text {7}$, $\text {F}_\text {a15}$ and $\text {F}_\text {a23}$; ENNA can share the best MEAN with EO, SOA, NNA, DE, MVO, WOA, BSA, CLNNA, and MLNNA in $\text {F}_\text {a14}$ and $\text {F}_\text {a26}$; ENNA can share the best MEAN with EO, NNA, DE, MVO, BSA, CLNNA, and MLNNA in $\text {F}_\text {a19}$; ENNA and the compared algorithms can share the best MEAN in $\text {F}_\text {a5}$, $\text {F}_\text {a12}$, $\text {F}_\text {a13}$, $\text {F}_\text {a16}$, $\text {F}_\text {a24}$, and $\text {F}_\text {a25}$. In addition, EO, DE, and MVO can share the best MEAN in $\text {F}_\text {a6}$. DE can obtain the best MEAN in $\text {F}_\text {a9}$ and $\text {F}_\text {a22}$. DE and EO can share the best MEAN in $\text {F}_\text {a27}$. EO can obtain the best MEAN in $\text {F}_\text {a28}$. CLNNA can achieve the best MEAN in $\text {F}_\text {a29}$. Thus, ENNA can get or share the best MEAN in 80 percent of the functions, which shows obvious advantages over the compared algorithms (Tables 3 and 4).

Table 5 presents the results of the Wilcoxon signed ranks test (the significance level is set to 0.05) of the solutions obtained by ENNA and the compared algorithms. In Table 5, the symbol is marked by ’+’, which indicates that ENNA can get better performance than the compared algorithm; the symbol is marked by ’-’, which indicates that ENNA can get worse performance than the compared algorithm; the symbol is marked by ’=’, which indicates that ENNA can get same performance than the compared algorithm. From Table 5, ENNA outperforms EO in half of test functions including $\text {F}_\text {a1}$, $\text {F}_\text {a2}$, $\text {F}_\text {a3}$, $\text {F}_\text {a4}$, $\text {F}_\text {a5}$, $\text {F}_\text {a8}$, $\text {F}_\text {a10}$, $\text {F}_\text {a11}$, $\text {F}_\text {a12}$, $\text {F}_\text {a16}$, $\text {F}_\text {a17}$, $\text {F}_\text {a18}$, $\text {F}_\text {a20}$, $\text {F}_\text {a21}$, and $\text {F}_\text {a30}$ while ENNA cannot compete with EO in 12 test functions, i.e., $\text {F}_\text {a6}$, $\text {F}_\text {a7}$, $\text {F}_\text {a13}$, $\text {F}_\text {a14}$, $\text {F}_\text {a15}$, $\text {F}_\text {a19}$, $\text {F}_\text {a22}$, $\text {F}_\text {a23}$, $\text {F}_\text {a24}$, $\text {F}_\text {a25}$, $\text {F}_\text {a27}$, and $\text {F}_\text {a28}$, and ENNA and EO have the same performance in three test functions, i.e., $\text {F}_\text {a9}$, $\text {F}_\text {a26}$, and $\text {F}_\text {a29}$. TSA can only beat ENNA in $\text {F}_\text {a24}$ while ENNA is superior to TSA in the rest 29 test functions. SOA only can get better performance than ENNA in $\text {F}_\text {a24}$, which has the same performance with ENNA in four test functions including $\text {F}_\text {a22}$, $\text {F}_\text {a25}$, $\text {F}_\text {a28}$, and $\text {F}_\text {a29}$, and can get worse performance than ENNA in the rest 25 test functions. NNA only outperforms ENNA in $\text {F}_\text {a5}$ and $\text {F}_\text {a27}$, and achieves the same performance with ENNA in $\text {F}_\text {a24}$ and $\text {F}_\text {a29}$, which cannot compete with ENNA in the rest 26 test functions. DE shows excellent competitiveness, which can get better performance than ENNA in nine test functions, i.e., $\text {F}_\text {a6}$, $\text {F}_\text {a8}$, $\text {F}_\text {a13}$, $\text {F}_\text {a15}$, $\text {F}_\text {a19}$, $\text {F}_\text {a22}$, $\text {F}_\text {a25}$, $\text {F}_\text {a27}$, and $\text {F}_\text {a28}$. DE also can get the same performance as ENNA in $\text {F}_\text {a14}$, $\text {F}_\text {a26}$, and $\text {F}_\text {a29}$ while DE still cannot search for the better results than ENNA in the rest sixty percent of test functions. Although MVO can obtain better performance than ENNA in $\text {F}_\text {a5}$, $\text {F}_\text {a6}$, $\text {F}_\text {a13}$, $\text {F}_\text {a15}$, $\text {F}_\text {a22}$, $\text {F}_\text {a24}$, $\text {F}_\text {a25}$, $\text {F}_\text {a27}$, and $\text {F}_\text {a28}$, it is inferior to ENNA in half of test functions. ENNA is superior to WOA in 26 test functions while WOA can only obtain better performance than ENNA in three test functions, i.e., $\text {F}_\text {a13}$, $\text {F}_\text {a14}$, and $\text {F}_\text {a24}$. BSA can beat ENNA in eight test functions, i.e., $\text {F}_\text {a7}$, $\text {F}_\text {a9}$, $\text {F}_\text {a13}$, $\text {F}_\text {a14}$, $\text {F}_\text {a15}$, $\text {F}_\text {a19}$, $\text {F}_\text {a22}$, and $\text {F}_\text {a24}$, which is inferior to ENNA in sixty percent of test functions. CLNNA and MLNNA can get better performance than ENNA in seven test functions (i.e., $\text {F}_\text {a5}$, $\text {F}_\text {a13}$, $\text {F}_\text {a14}$, $\text {F}_\text {a24}$, and $\text {F}_\text {a25}$) and three test functions (i.e., $\text {F}_\text {a5}$, $\text {F}_\text {a27}$, and $\text {F}_\text {a28}$), respectively. However, both CLNNA and MLNNA are inferior to ENNA in two-thirds of test functions. That is, according to the results of the Wilcoxon signed ranks test (the significance level is set to 0.05), ENNA outperforms each of the comparison algorithms on at least half of the test functions, which fully demonstrates the performance advantages of ENNA over the comparison algorithms in terms of solving the CEC 2014 test suite.

**Fig. 4:**
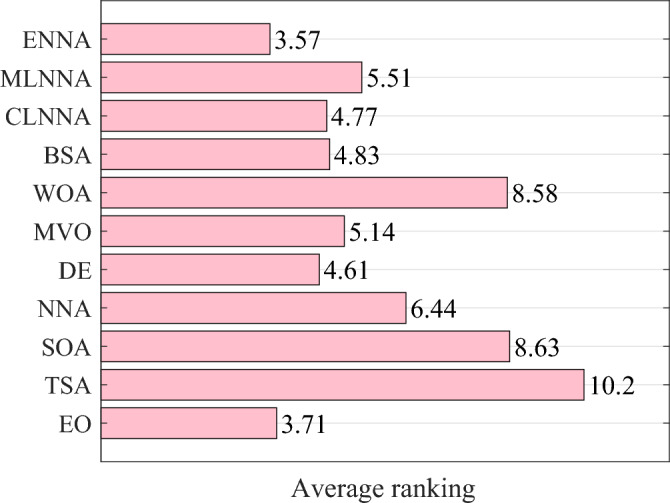
The average rankings of all algorithms on the CEC 2014 test suite based on the Friedman test.

**Fig. 5:**
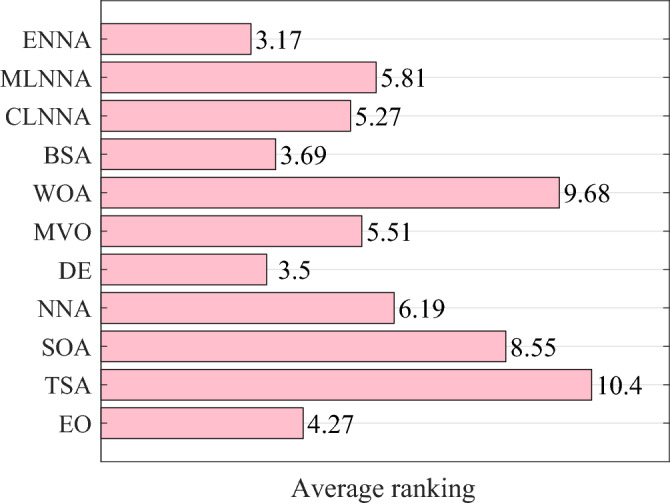
The average rankings of all algorithms on the CEC 2020 test suite based on the Friedman test.

**Fig. 6:**
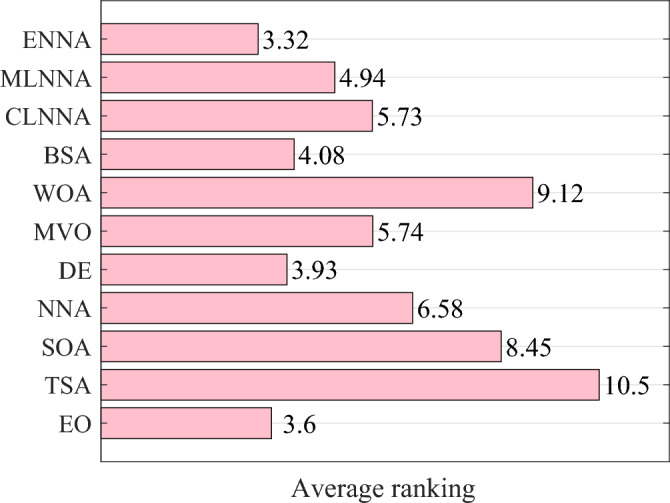
The average rankings of all algorithms on the CEC 2022 test suite based on the Friedman test.

**Fig. 7:**
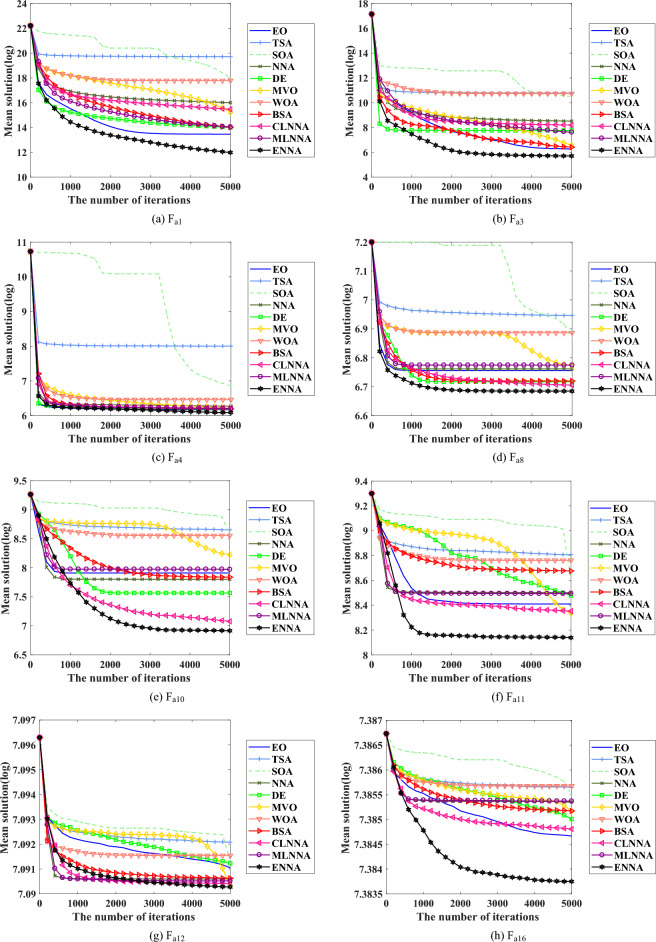
Some typical convergence curves obtained by ENNA and the compared algorithms on the CEC 2014 test suite.

Figure 4 shows the average ranking of all algorithms on the CEC 2014 test suite based on the Friedman test. According to the Friedman test, a small average ranking means a better performance. Thus, from Fig. 4, all algorithms can be sorted from the best to the worst as follows: ENNA, EO, DE, CLNNA, BSA, MVO, MLNNA, NNA, SOA, WOA, and TSA. Clearly, ENNA is the best of all algorithms in solving the CEC 2014 test suite (Figs. 5 and 6). Some typical convergence curves obtained by ENNA and the compared algorithms in the CEC 2014 test suite have been shown in Fig. 7. As shown in Fig. 7, ENNA exhibits more significant convergence advantages than the compared algorithms in $\text {F}_\text {a1}$, $\text {F}_\text {a2}$, $\text {F}_\text {a8}$, $\text {F}_\text {a10}$, $\text {F}_\text {a11}$, and $\text {F}_\text {a16}$, and can search for better solutions with fewer iterations compared to the compared algorithms. In $\text {F}_\text {a4}$ and $\text {F}_\text {a12}$, although ENNA does not show a significant convergence advantage over the comparison algorithm, it is still able to achieve better solutions compared to the comparison algorithm.

### Comparison on CEC 2020 test suite

This section is to compare the performance between ENNA and the compared algorithms on 10 benchmark functions with four types from CEC 2020 test suite^49^, which include one unimodal functions (i.e., $\text {F}_\text {b1}$), three simple multimodal functions (i.e., $\text {F}_\text {b2}$, $\text {F}_\text {b3}$, and $\text {F}_\text {b4}$), three hybrid functions (i.e., $\text {F}_\text {b5}$, $\text {F}_\text {b6}$, and $\text {F}_\text {b7}$), and three composition functions ($\text {F}_\text {b8}$, $\text {F}_\text {b9}$, and $\text {F}_\text {b10}$). In this experiment, the dimension of each function is set to 20; the population size of each algorithm is set to 30; the maximum number of iterations is set to 5,000; the number of independent repeated experiments is set to 50. The other control parameters of the compared algorithms are extracted from the corresponding original references.

The experimental results from ENNA and the compared algorithms on the CEC 2020 test suite are shown in Table 3. In Table 3, MEAN and STD represent the mean value and standard deviation, respectively. As can be seen from Table 3, ENNA can get the best MEAN in half of test functions including $\text {F}_\text {b1}$, $\text {F}_\text {b3}$, $\text {F}_\text {b4}$, $\text {F}_\text {b8}$, and $\text {F}_\text {b10}$. In addition, ENNA can share the best MEAN with BSA in $\text {F}_\text {b2}$; ENNA can share the best MEAN with the other 10 algorithms in $\text {F}_\text {b5}$; ENNA can share the best MEAN with EO, NNA, DE, MVO, BSA, WOA, CLNNA, and MLNNA in $\text {F}_\text {b7}$. In addition, DE, EO, and MVO can obtain the best MEAN in $\text {F}_\text {b6}$, and DE can achieve the best MEAN in $\text {F}_\text {b9}$. That is, ENNA can get or share the best MEAN in 80 percent of the test functions, which is obviously superior to the compared algorithms.

Table 6 displays the results of the Wilcoxon signed ranks test (the significance level is set to 0.05) of the solutions obtained by ENNA and the compared algorithms on the CEC 2020 test suite. In Table 6, the symbol is marked by ’+’, which indicates that ENNA can get better performance than the compared algorithm; the symbol is marked by ’-’, which indicates that ENNA can get worse performance than the compared algorithm; the symbol is marked by ’=’, which indicates that ENNA can get same performance than the compared algorithm. From Table 6, ENNA outperforms TSA and WOA in all test functions. EO can only get better performance than ENNA in $\text {F}_\text {b9}$ while ENNA can beat EO in six test functions, i.e., $\text {F}_\text {b1}$, $\text {F}_\text {b2}$, $\text {F}_\text {b3}$, $\text {F}_\text {b5}$, $\text {F}_\text {b6}$, and $\text {F}_\text {b7}$. SOA outperforms ENNA in $\text {F}_\text {b9}$ while it is inferior to ENNA in the rest nine test functions. NNA can get better performance than ENNA in$\text {F}_\text {b5}$ and the same performance with ENNA in $\text {F}_\text {b8}$, $\text {F}_\text {b9}$, and $\text {F}_\text {b10}$, which has no advantages over ENNA in $\text {F}_\text {b1}$, $\text {F}_\text {b2}$, $\text {F}_\text {b3}$, $\text {F}_\text {b4}$, $\text {F}_\text {b6}$, and $\text {F}_\text {b7}$. Although DE has advantages over ENNA in $\text {F}_\text {b3}$, $\text {F}_\text {b9}$, and $\text {F}_\text {b10}$, it is inferior to ENNA in $\text {F}_\text {b1}$, $\text {F}_\text {b2}$, $\text {F}_\text {b5}$, $\text {F}_\text {b6}$, and $\text {F}_\text {b7}$. ENNA outperforms MVO in 80% of test functions including $\text {F}_\text {b1}$, $\text {F}_\text {b2}$, $\text {F}_\text {b3}$, $\text {F}_\text {b4}$, $\text {F}_\text {b5}$, $\text {F}_\text {b6}$, $\text {F}_\text {b7}$, and $\text {F}_\text {b8}$. BSA shows excellent optimization performance, which has the same performance with ENNA in five test functions, i.e., $\text {F}_\text {b3}$, $\text {F}_\text {b4}$, $\text {F}_\text {b5}$, $\text {F}_\text {b7}$, and $\text {F}_\text {b10}$. However, ENNA still gets better performance than BSA in three test functions (i.e., $\text {F}_\text {b2}$, $\text {F}_\text {b6}$, and $\text {F}_\text {b8}$). Both CLNNA and MLNNA are inferior to ENNA in 70% of the test functions including $\text {F}_\text {b1}$, $\text {F}_\text {b2}$, $\text {F}_\text {b3}$, $\text {F}_\text {b4}$, $\text {F}_\text {b5}$, $\text {F}_\text {b6}$, and $\text {F}_\text {b7}$. Thus, according to the results of the Wilcoxon signed ranks test (the significance level is set to 0.05), ENNA outperforms the compared algorithms in terms of solving the CEC 2020 test suite.

**Fig. 8:**
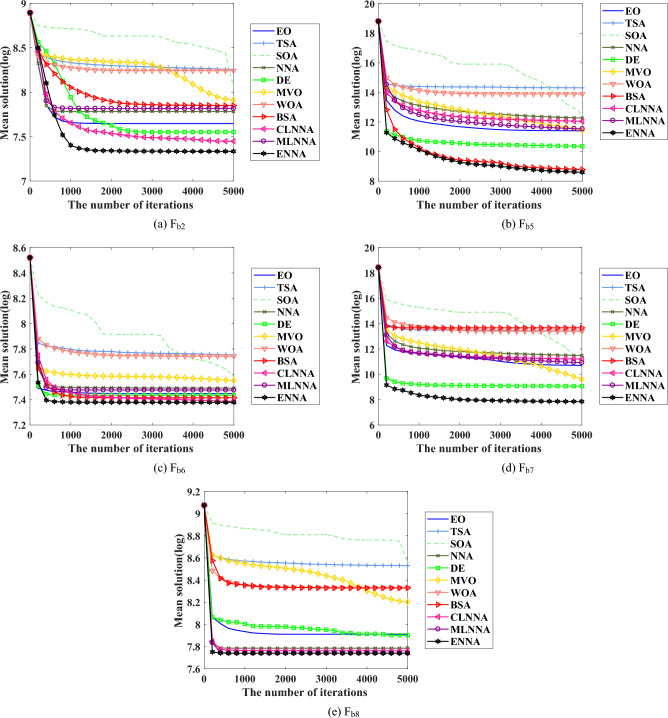
Some typical convergence curves obtained by ENNA and the compared algorithms on the CEC 2020 test suite.

Fig. 5 presents the average ranking of all algorithms on the CEC 2014 test suite based on the Friedman test. According to the Friedman test, a small average ranking means a better performance. Looking at Fig. 5, all algorithms can be sorted from the best to the worst as follows: ENNA, DE, BSA, EO, CLNNA, MVO, MLNNA, NNA, SOA, WOA, and TSA. That is, ENNA is the best of all algorithms. Some typical convergence curves obtained by ENNA and the compared algorithms in the CEC 2020 test suite have been shown in Fig. 8. As shown in Fig. 8, ENNA demonstrates a significant convergence advantage over the compared algorithms in both $\text {F}_\text {b2}$ and $\text {F}_\text {b7}$, and is able to search for better solutions with fewer iterations compared to the 10 compared algorithms. Although ENNA does not demonstrate a significant convergence advantage over the comparison algorithms in $\text {F}_\text {b5}$, $\text {F}_\text {b6}$, and $\text {F}_\text {b8}$, it still has a slight advantage in solution quality compared to the comparison algorithms. It is worth mentioning that the CEC 2020 test suite has a total of 10 functions, and ENNA outperforms the compared algorithms in terms of convergence performance in all 5 functions, demonstrating excellent global search performance.

### Comparison on CEC 2022 test suite

This section is to compare the performance between ENNA and the compared algorithms on 12 benchmark functions with four types from CEC 2022 test suite^50^, which include one unimodal functions (i.e., $\text {F}_\text {c1}$), four simple multimodal functions (i.e., $\text {F}_\text {c2}$, $\text {F}_\text {c3}$, $\text {F}_\text {c4}$, and $\text {F}_\text {c5}$), three hybrid functions (i.e., $\text {F}_\text {c6}$, $\text {F}_\text {c7}$, and $\text {F}_\text {c8}$), and four composition functions ($\text {F}_\text {c9}$, $\text {F}_\text {c10}$, $\text {F}_\text {c11}$, and $\text {F}_\text {c12}$). In this experiment, the dimension of each function is set at 20; the population size of each algorithm is set to 30; the maximum number of iterations is set to 5,000; the number of independent repeated experiments is set to 50. The other control parameters of the compared algorithms are extracted from the corresponding original references.

The experimental results from ENNA and the compared algorithms on the CEC 2022 test suite have been presented in Table 4. In Table 4, MEAN and STD represent the mean value and standard deviation, respectively. From Table 4, ENNA can get the best MEAN in half of the test functions, i.e., $\text {F}_\text {c1}$, $\text {F}_\text {c3}$, $\text {F}_\text {c4}$, $\text {F}_\text {c8}$, $\text {F}_\text {c10}$, and $\text {F}_\text {c11}$. In addition, ENNA can share the best MEAN with BSA in $\text {F}_\text {c2}$; ENNA and the other 10 algorithms can share the best MEAN in $\text {F}_\text {c5}$ and $\text {F}_\text {c12}$; ENNA, NNA, DE, MVO, WOA, BSA, CLNNA, and MLNNA can share the best MEAN in $\text {F}_\text {c7}$. In addition, EO, DE, and MVO can share the best MEAN in $\text {F}_\text {c6}$, and DE can obtain the best MEAN in $\text {F}_\text {c9}$. That is, ENNA can obtain or share the best MEAN in more than 80 percent of the test functions.

Table 7 shows the results of the Wilcoxon signed ranks test (the significance level is set to 0.05) of the solutions obtained by ENNA and the compared algorithms in the CEC 2020 test suite. In Table 7, the symbol is marked by ’+’, which indicates that ENNA can get better performance than the compared algorithm; the symbol is marked by ’-’, which indicates that ENNA can get worse performance than the compared algorithm; the symbol is marked by ’=’, which indicates that ENNA can get same performance than the compared algorithm. As shown in Table 7, ENNA outperforms TSA, and WOA in all test functions. ENNA also has obvious advantages over SOA, NNA, CLNNA, MVO, and MLNNA. EO is superior to ENNA in four test functions (i.e., $\text {F}_\text {c3}$, $\text {F}_\text {c5}$, $\text {F}_\text {c9}$, and $\text {F}_\text {c12}$) while it is inferior to ENNA in six test functions, i.e., $\text {F}_\text {c1}$, $\text {F}_\text {c2}$, $\text {F}_\text {c6}$, $\text {F}_\text {c7}$, $\text {F}_\text {c8}$, and $\text {F}_\text {c10}$. DE shows better performance than ENNA in four test functions (i.e., $\text {F}_\text {c3}$, $\text {F}_\text {c4}$, $\text {F}_\text {c5}$, and $\text {F}_\text {c12}$) while it is inferior to ENNA in six test functions, i.e., $\text {F}_\text {c1}$, $\text {F}_\text {c2}$, $\text {F}_\text {c6}$, $\text {F}_\text {c9}$, $\text {F}_\text {c10}$, and $\text {F}_\text {c11}$. BSA can achieve better performance than ENNA in three test functions (i.e., $\text {F}_\text {c3}$, $\text {F}_\text {c4}$, and $\text {F}_\text {c5}$) while it cannot compete with ENNA in nearly half of the test functions including $\text {F}_\text {c1}$, $\text {F}_\text {c2}$, $\text {F}_\text {c6}$, $\text {F}_\text {c7}$, and $\text {F}_\text {c10}$. Thus, according to the results of the Wilcoxon signed ranks test (the significance level is set to 0.05), ENNA outperforms the compared algorithms in terms of solving the CEC 2022 test suite.

**Fig. 9:**
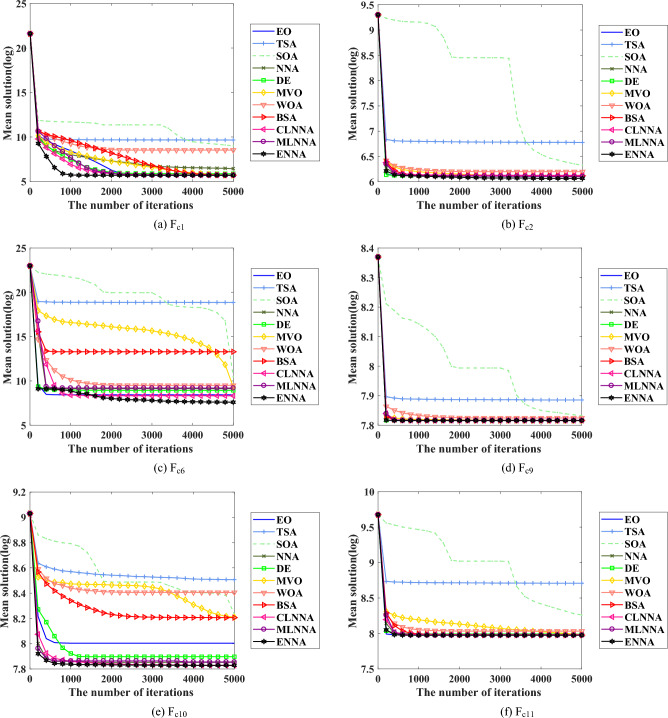
Some typical convergence curves obtained by ENNA and the compared algorithms on the CEC 2022 test suite.

Fig. 6 shows the average rankings of all algorithms in the CEC 2022 test suite based on the Friedman test. According to the Friedman test, a small average ranking means a better performance. Looking at Fig. 6, all algorithms can be sorted from the best to the worst as follows: ENNA, EO, DE, BSA, MLNNA, CLNNA, MVO, NNA, SOA, WOA, and TSA. That is, ENNA is the best of all algorithms. Some typical convergence curves obtained by ENNA and the compared algorithms in the CEC 2022 test suite have been shown in Fig. 9. As shown in Fig. 9, ENNA exhibits a significant convergence advantage over the comparison algorithm in $\text {F}_\text {c6}$, as it can find significantly better solutions with fewer iterations compared to the comparison algorithm. Although ENNA does not demonstrate a significant convergence advantage over the comparison algorithms in $\text {F}_\text {c1}$, $\text {F}_\text {c2}$, $\text {F}_\text {c9}$, $\text {F}_\text {c10}$, and $\text {F}_\text {c11}$, it still has a slight advantage in solution quality compared to the comparison algorithms. In addition, the CEC 2022 test suite has a total of 12 functions, and as shown in Fig. 9, ENNA outperforms the compared algorithms on half of the functions, demonstrating excellent convergence performance.

## Parameter extraction of PV models

This section is to investigate the performance of ENNA in the parameter extraction of three different types of PV models, which is divided into three subsections. Firstly, Section presents the three PV models. Then, Section compares the performance differences between the proposed ENNA and the compared algorithms. Lastly, according to the experimental results shown in Section, Section discusses the validity of the improved strategies.

**Fig. 10:**
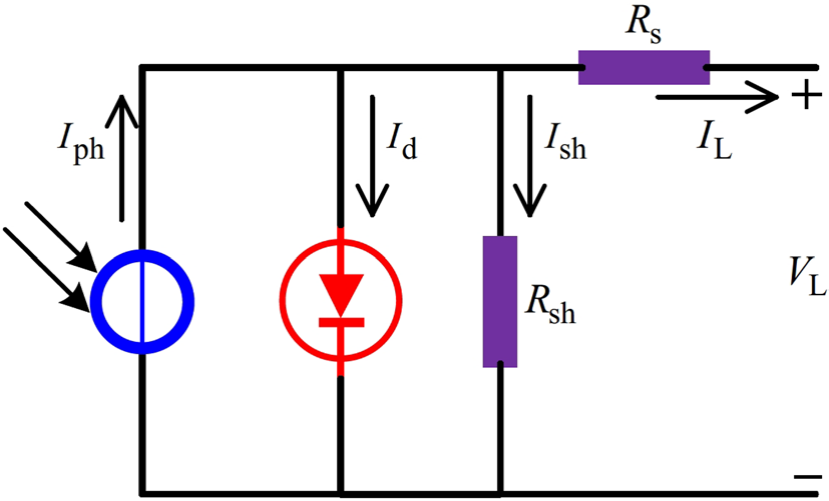
The circuit structure of SDM.

**Fig. 11:**
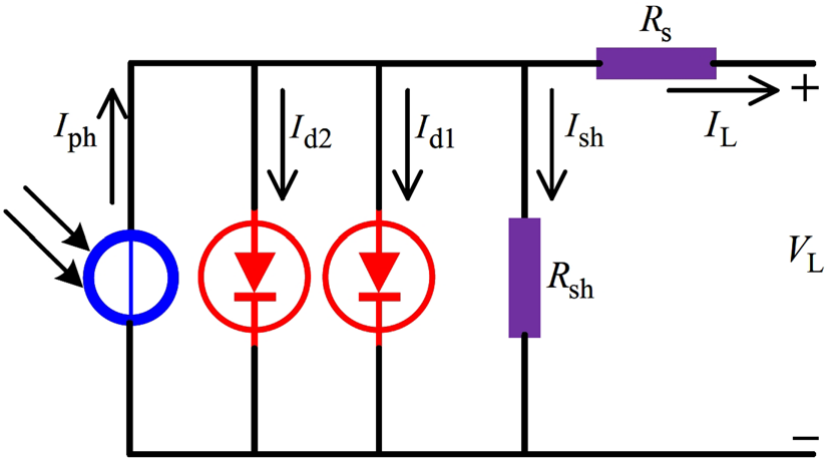
The circuit structure of DDM.

**Fig. 12:**
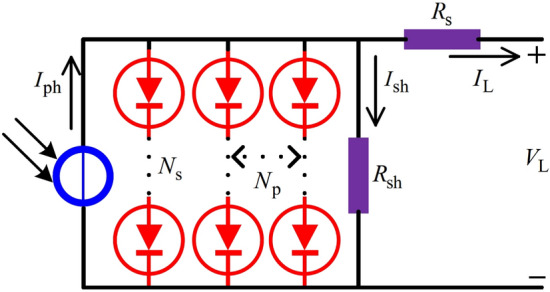
The circuit structure of the PV module.

**Table 8:** The measured voltage and current of a 57 mm diameter silicon solar cell at 33$\phantom{0}^{\circ }$C^60^.

Case	$V_\text {m}$	$I_\text {m}$
Point 1	−0.2057	0.7640
Point 2	−0.1291	0.7620
Point 3	−0.0588	0.7605
Point 4	0.0057	0.7605
Point 5	0.0646	0.7600
Point 6	0.1185	0.7590
Point 7	0.1678	0.7570
Point 8	0.2132	0.7570
Point 9	0.2545	0.7555
Point 10	0.2924	0.7540
Point 11	0.3269	0.7505
Point 12	0.3585	0.7465
Point 13	0.3873	0.7385
Point 14	0.4137	0.7280
Point 15	0.4373	0.7065
Point 16	0.4590	0.6755
Point 17	0.4784	0.6320
Point 18	0.4960	0.5730
Point 19	0.5119	0.4990
Point 20	0.5265	0.4130
Point 21	0.5398	0.3165
Point 22	0.5521	0.2120
Point 23	0.5633	0.1035
Point 24	0.5736	−0.0100
Point 25	0.5833	−0.1230
Point 26	0.5900	−0.2100

**Table 9:** The measured voltage and current of the Photowatt-PWP201 with 36 cells at 45$\phantom{0}^{\circ }$C^61^.

Case	$V_\text {m}$	$I_\text {m}$
Point 1	0.1248	1.0315
Point 2	1.8093	1.0300
Point 3	3.3511	1.0260
Point 4	4.7622	1.0220
Point 5	6.0538	1.0180
Point 6	7.2364	1.0155
Point 7	8.3189	1.0140
Point 8	9.3097	1.0100
Point 9	10.2163	1.0035
Point 10	11.0449	0.9880
Point 11	11.8018	0.9630
Point 12	12.4929	0.9255
Point 13	13.1231	0.8725
Point 14	13.6983	0.8075
Point 15	14.2221	0.7265
Point 16	14.6995	0.6345
Point 17	15.1346	0.5345
Point 18	15.5311	0.4275
Point 19	15.8929	0.3185
Point 20	16.2229	0.2085
Point 21	16.5241	0.1010
Point 22	16.7987	−0.0080
Point 23	17.0499	−0.1110
Point 24	17.2793	−0.2090
Point 25	17.4885	−0.3030

**Table 10:** The upper and lower boundaries of the unknown parameters^62^.

Parameter	SDM		DDM		PVM	
	UB	LB	UB	LB	UB	LB
$I_\text {ph} (\text {A})$	1	0	1	0	1	0
$I_\text {d} (\text {A})$	1	0	-	-	1	0
$I_\text {d1} (\text {A})$	-	-	1	0	-	-
$I_\text {d2} (\text {A})$	-	-	1	0	-	-
$R_\text {s} (\text {ou})$	0.5	0	0.5	0	0.5	0
$R_\text {sh} (\text {ou})$	100	0	100	0	100	0
*n*	2	1	-	-	2	1
$n_1$	-	-	2	1	-	-
$n_2$	-	-	2	1	-	-

### Problem statement

This section describes the mathematical models of the considered SDM, DDM, and PV module (PVM).

As presented in Fig. 10, SDM is a very popular PV model, which consists of a series resistance $(R_\text {s})$, a shunt resistance $(R_\text {sh})$, a diode, and a photo-generated controlled current source $(I_\text {ph})$. In Fig. 10, $I_\text {d}$ is the current flowing through the diode and $I_\text {sh}$ is the current flowing through $R_\text {sh}$. The output current $I_\text {L}$ of SDM can be computed by^62–67^:

11a $$\begin{aligned}&I_\text {L}=I_\text {ph}-I_\text {sh}-I_\text {d}, \end{aligned}$$

11b $$\begin{aligned}&I_\text {sh}=\frac{V_\text {L}+R_\text {s}\cdot I_\text {L}}{R_\text {sh}}, \end{aligned}$$

11c $$\begin{aligned}&I_\text {d}=I_\text {sd}\cdot \left[ \text {exp}\left( \frac{V_\text {L}\cdot q+R_\text {s}\cdot I_\text {L}\cdot q}{n\cdot T \cdot k}\right) -1\right] , \end{aligned}$$

where $I_\text {sd}$ is the reverse saturation current of the diode, *n* is the ideal factor, *k* is the Boltzmann constant $(k=1.380649\times 10^{-23}J/K)$, *q* is the electron charge $(q=1.602176634\times 10^{-19}C)$, and *T* is the current temperature in kelvin. From Eq. (11c), there are five unknown parameters that need to be estimated, which are $I_\text {ph}$, $I_\text {sd}$, $R_\text {s}$, $R_\text {sh}$, and *n*.

From Fig. 11, DDM has one more diode than SDM, which consists of a series resistance $(R_\text {s})$, a shunt resistance $(R_\text {sh})$, two diodes, and a photo-generated controlled current source $(I_\text {ph})$. The output current $I_\text {L}$ of DDM can be computed by^62–67^:

12a $$\begin{aligned} I_\text {L}&=I_\text {ph}-I_\text {sh}-I_\text {d1}-I_\text {d2}, \end{aligned}$$

12b $$\begin{aligned} I_\text {sh}&=\frac{V_\text {L}+R_\text {s}\cdot I_\text {L}}{R_\text {sh}}, \end{aligned}$$

12c $$\begin{aligned} \qquad I_\text {d1}&=I_\text {sd1}\cdot \left[ \text {exp}\left( \frac{V_\text {L}\cdot q+R_\text {s}\cdot I_\text {L}\cdot q}{n_1\cdot T \cdot k}\right) -1\right] , \end{aligned}$$

12d $$\begin{aligned} \qquad I_\text {d2}&=I_\text {sd2}\cdot \left[ \text {exp}\left( \frac{V_\text {L}\cdot q+R_\text {s}\cdot I_\text {L}\cdot q}{n_2\cdot T \cdot k}\right) -1\right] , \end{aligned}$$

where $I_\text {d1}$ is the current flowing through diode 1, $I_\text {d2}$ is the current flowing through diode 2, $n_1$ is the ideal factor of diode 1, $n_2$ is the ideal factor of diode 2, $I_\text {sd1}$ is the reverse saturation current of diode 1, and $I_\text {sd2}$ is the reverse saturation current of diode 2. From Eq. (12d), there are seven unknown parameters that need to be estimated, which are $I_\text {ph}$, $I_\text {sd1}$, $I_\text {sd2}$, $R_\text {s}$, $R_\text {sh}$, $n_1$, and $n_2$.

As presented in Fig. 12, there are $N_\text {s}\times N_\text {p}$ diodes are connected in parallel or series within the structure of PVM. The output current $I_\text {L}$ of PVM can be computed by^62–65,68^:

13a $$\begin{aligned}&I_\text {L}=N_\text {p}\times I_\text {ph}-N_\text {p}\times I_\text {sh}-N_\text {p}\times I_\text {d}, \end{aligned}$$

13b $$\begin{aligned}&I_\text {sh}=\frac{\frac{V_\text {L}}{N_\text {s}}+R_\text {s}\cdot \frac{I_\text {L}}{N_\text {p}}}{R_\text {sh}}, \end{aligned}$$

13c $$\begin{aligned}&I_\text {d}=I_\text {sd}\cdot \left[ \text {exp}\left( \frac{V_\text {L}\cdot \frac{I_\text {L}}{N_\text {s}}\cdot q+R_\text {s}\cdot \frac{I_\text {L}}{N_\text {p}}\cdot q}{n\cdot T \cdot k}\right) -1\right] . \end{aligned}$$

From Eq. (13c), there are five unknown parameters that need to be estimated, which are $I_\text {ph}$, $I_\text {sd}$, $R_\text {s}$, $R_\text {sh}$, and *n*.

As described in Eqs. (11-13), the unknown parameters in the three PV models are highly correlated with the performance of PV models. To accurately extract the unknown parameters, the parameter estimation problem of the PV model is usually transformed into an optimization problem, whose objective function is the root mean square error (RMSE) based on the measured data and experimental data^62–65,67–71^, namely:

14a $$\begin{aligned}&\text {Min}(\text {RMSE})=f(\boldsymbol{x})=\sqrt{\frac{1}{N_\text {c}}\sum _{ i=0}^{N_\text {c}}\left[ I^\text {m}_{\text {L},i}-I^\text {e}_{\text {L},i}\right] ^2}, \end{aligned}$$

14b $$\begin{aligned}&I^\text {e}_{\text {L},i}=g\left( \boldsymbol{x},V^\text {m}_{\text {L},i}\right) , \end{aligned}$$

where $\boldsymbol{x}$ is the set of the unknown parameters, $N_\text {c}$ is the number of points in the measured data, $I^\text {m}_{\text {L},i}$ is the measured current at point *i*, $V^\text {m}_{\text {L},i}$ is the measured voltage at point *i*, $I^\text {e}_{\text {L},i}$ is the estimated current at point *i* and $g(\cdot )$ is a function of computing $I^\text {e}_{\text {L},i}$. Specifically, $g(\cdot )$ can be obtained by Eq. (11), Eq. (12), and Eq. (13) for SDM, DDM, and PVM, respectively. In addition, the measured data of SDM and DDM are extracted from France solar cells as shown in Table 8, which is measured on a 57 mm diameter silicon solar cell at 33$\phantom{0}^{\circ }$C^60,62^; the measured data of PDM is extracted from the Photowatt-PWP201 with 36 cells at 45$\phantom{0}^{\circ }$C as shown in Table 9^72^. Table 10 presents the lower and upper boundaries of the unknown parameters in the considered PV models. In Table 10, UB and LB represent the upper boundary and the lower boundary, respectively. The smaller the value of RMSE is, the smaller the difference between the experimental data and the benchmark data will be, and it also indicates that the estimated parameters are closer to the true parameters. So, the algorithm with a smaller RMSE obtained is one with higher parameter estimation accuracy, and the estimated parameters it acquires are more suitable for constructing real PV models.

**Table 11:** The statistical results obtained by the 11 algorithms on the SDM.

Algorithm	MAX	MEAN	MEDIAN	MIN	STD
EO	0.03815132	0.00214091	0.00146451	0.00098619	5.21E-03
TSA	0.04525377	0.00724087	0.00390740	0.00170553	1.01E-02
SOA	0.22286192	0.09917866	0.04691879	0.00284886	8.67E-02
NNA	0.00242838	0.00213802	0.00232427	0.00098619	4.14E-04
DE	0.03815132	0.00207693	0.00132226	0.00098602	5.22E-03
MVO	0.01237734	0.00455950	0.00338997	0.00130382	2.91E-03
WOA	0.08007894	0.01707405	0.00483100	0.00119521	2.04E-02
BSA	0.00110477	0.00103558	0.00103309	0.00099656	2.52E-05
CLNNA	0.00388037	0.00189675	0.00175532	0.00102053	6.11E-04
MLNNA	0.00233204	0.00195073	0.00211015	0.00098728	3.87E-04
ENNA	0.00098602	0.00098602	0.00098602	0.00098602	2.73E-17

**Table 12:** The statistical results obtained by the 11 algorithms on the DDM.

Algorithm	MAX	MEAN	MEDIAN	MIN	STD
EO	0.002448043	0.001446033	0.001463006	0.000982940	3.44E-04
TSA	0.044818220	0.007494715	0.004687766	0.001477534	9.77E-03
SOA	0.222862088	0.082610262	0.045984111	0.004850166	8.06E-02
NNA	0.003864797	0.002299850	0.002375104	0.000984631	7.73E-04
DE	0.002282192	0.001260141	0.001143816	0.000983458	3.18E-04
MVO	0.008121311	0.003965896	0.003988999	0.001193188	1.52E-03
WOA	0.047611497	0.014025126	0.005485817	0.001367805	1.53E-02
BSA	0.033391870	0.001805438	0.001100642	0.000987992	4.56E-03
CLNNA	0.006238953	0.002231221	0.002051399	0.001136820	1.04E-03
MLNNA	0.003650084	0.002335322	0.002385991	0.001055564	5.30E-03
ENNA	0.000997408	0.000984296	0.000982972	0.000982485	2.78E-06

**Table 13:** The statistical results obtained by the 11 algorithms on the PVM.

Algorithm	MAX	MEAN	MEDIAN	MIN	STD
EO	0.27425078	0.03527130	0.00249573	0.00243065	8.91E-02
TSA	0.07300405	0.00555379	0.00386741	0.00269771	9.80E-03
SOA	0.78391195	0.24943161	0.27425097	0.00272699	1.12E-01
NNA	0.27425078	0.01374791	0.00259281	0.00243953	5.37E-02
DE	0.27678741	0.02429461	0.00243079	0.00242507	7.46E-02
MVO	0.27425084	0.00843770	0.00272971	0.00246073	3.84E-02
WOA	0.78391195	0.12858299	0.05598757	0.00254225	1.56E-01
BSA	0.27425078	0.02019661	0.00243520	0.00242792	6.56E-02
CLNNA	0.27425080	0.11243296	0.00477950	0.00245611	1.34E-01
MLNNA	0.00561127	0.00267575	0.00258352	0.00242828	5.21E-04
ENNA	0.27425078	0.00786159	0.00242507	0.00242507	3.84E-02

**Fig. 13:**
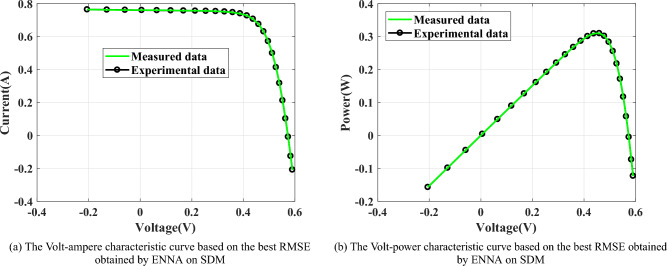
The characteristic curves obtained by ENNA on SDM.

**Fig. 14:**
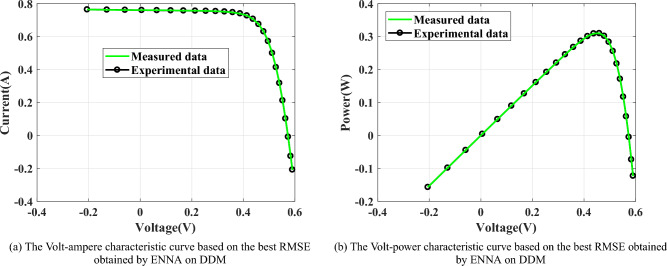
The characteristic curves obtained by ENNA on DDM.

**Fig. 15:**
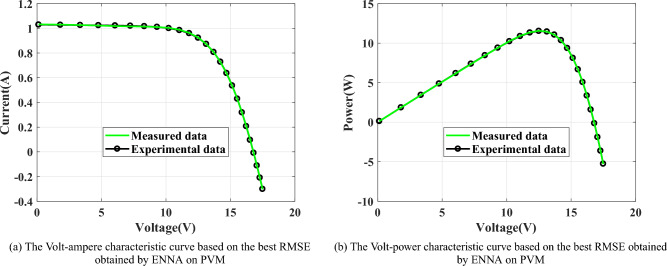
The characteristic curves obtained by ENNA on PVM.

**Fig. 16:**
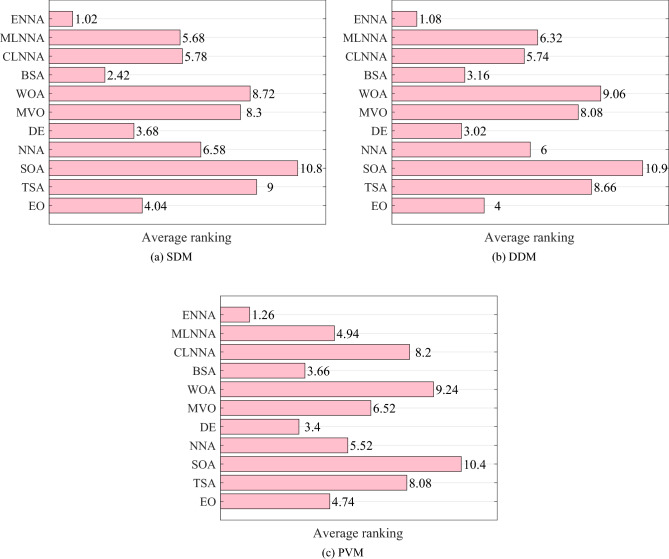
The average rankings of all algorithms in the SDM, DDM, and PVM.

**Fig. 17:**
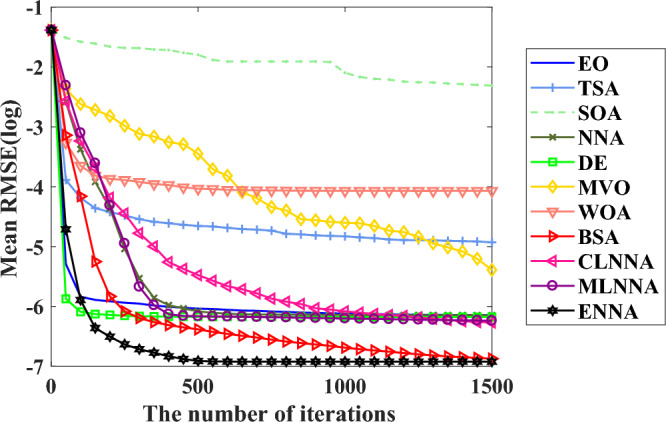
Convergence curves of the applied algorithms on SDM.

**Fig. 18:**
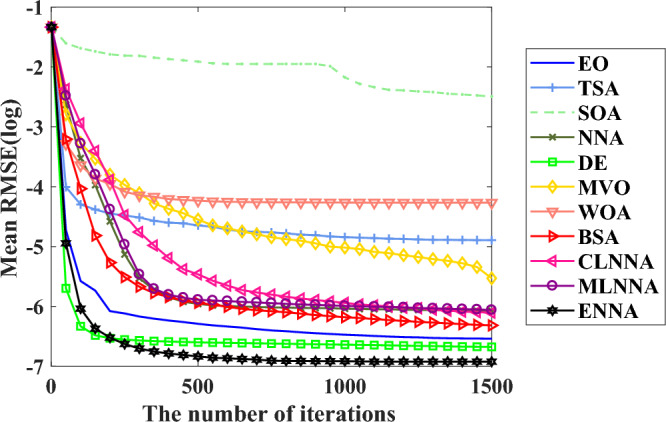
Convergence curves of the applied algorithms on DDM.

**Fig. 19:**
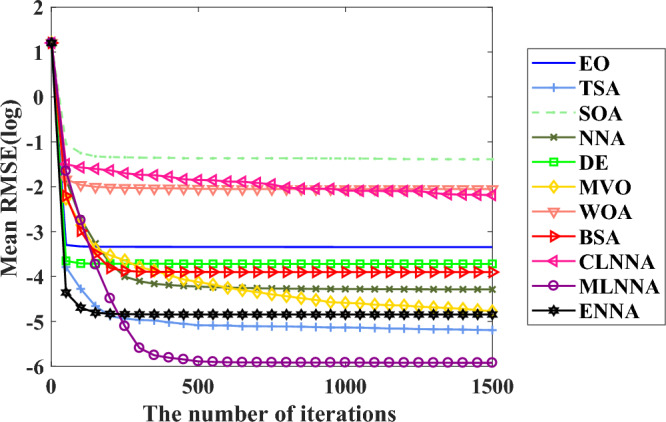
Convergence curves of the applied algorithms on PVM.

**Table 14:** The estimated current corresponding to the obtained best RMSE by the 11 algorithms on the SDM.

Case	$V_\text {m}$	$I_\text {m}$	$I_\text {e}$										
			EO	TSA	SOA	NNA	DE	MVO	WOA	BSA	CLNNA	MLNNA	ENNA
Point 1	−0.2057	0.7640	0.7641	0.7639	0.7697	0.7640	0.7641	0.7640	0.7626	0.7637	0.7643	0.7639	0.7641
Point 2	−0.1291	0.7620	0.7627	0.7626	0.7670	0.7626	0.7627	0.7626	0.7614	0.7624	0.7628	0.7626	0.7627
Point 3	−0.0588	0.7605	0.7614	0.7615	0.7644	0.7613	0.7614	0.7613	0.7603	0.7612	0.7614	0.7613	0.7614
Point 4	0.0057	0.7605	0.7602	0.7604	0.7621	0.7601	0.7602	0.7601	0.7593	0.7600	0.7601	0.7601	0.7602
Point 5	0.0646	0.7600	0.7591	0.7594	0.7600	0.7590	0.7591	0.7591	0.7584	0.7590	0.7590	0.7590	0.7591
Point 6	0.1185	0.7590	0.7581	0.7585	0.7580	0.7580	0.7580	0.7581	0.7575	0.7580	0.7579	0.7580	0.7580
Point 7	0.1678	0.7570	0.7571	0.7577	0.7562	0.7571	0.7571	0.7572	0.7567	0.7571	0.7569	0.7571	0.7571
Point 8	0.2132	0.7570	0.7562	0.7569	0.7545	0.7562	0.7561	0.7563	0.7559	0.7562	0.7559	0.7562	0.7561
Point 9	0.2545	0.7555	0.7551	0.7559	0.7527	0.7551	0.7551	0.7553	0.7550	0.7552	0.7548	0.7551	0.7551
Point 10	0.2924	0.7540	0.7537	0.7546	0.7507	0.7537	0.7537	0.7539	0.7537	0.7538	0.7534	0.7537	0.7537
Point 11	0.3269	0.7505	0.7514	0.7526	0.7479	0.7514	0.7514	0.7518	0.7516	0.7515	0.7511	0.7514	0.7514
Point 12	0.3585	0.7465	0.7474	0.7488	0.7435	0.7474	0.7474	0.7480	0.7477	0.7475	0.7471	0.7474	0.7474
Point 13	0.3873	0.7385	0.7401	0.7419	0.7361	0.7401	0.7401	0.7410	0.7406	0.7402	0.7400	0.7402	0.7401
Point 14	0.4137	0.7280	0.7274	0.7296	0.7234	0.7274	0.7274	0.7286	0.7281	0.7274	0.7274	0.7274	0.7274
Point 15	0.4373	0.7065	0.7070	0.7095	0.7033	0.7070	0.7070	0.7085	0.7078	0.7069	0.7072	0.7070	0.7070
Point 16	0.4590	0.6755	0.6753	0.6781	0.6721	0.6753	0.6753	0.6770	0.6763	0.6751	0.6757	0.6753	0.6753
Point 17	0.4784	0.6320	0.6307	0.6335	0.6283	0.6308	0.6308	0.6325	0.6317	0.6306	0.6312	0.6307	0.6308
Point 18	0.4960	0.5730	0.5719	0.5742	0.5703	0.5719	0.5719	0.5734	0.5728	0.5717	0.5724	0.5719	0.5719
Point 19	0.5119	0.4990	0.4996	0.5011	0.4988	0.4996	0.4996	0.5006	0.5002	0.4994	0.4999	0.4996	0.4996
Point 20	0.5265	0.4130	0.4136	0.4142	0.4134	0.4136	0.4136	0.4139	0.4139	0.4135	0.4138	0.4136	0.4136
Point 21	0.5398	0.3165	0.3175	0.3171	0.3177	0.3175	0.3175	0.3171	0.3175	0.3175	0.3174	0.3175	0.3175
Point 22	0.5521	0.2120	0.2122	0.2110	0.2124	0.2121	0.2122	0.2112	0.2119	0.2122	0.2118	0.2122	0.2122
Point 23	0.5633	0.1035	0.1023	0.1008	0.1022	0.1022	0.1023	0.1011	0.1018	0.1024	0.1019	0.1023	0.1023
Point 24	0.5736	−0.0100	−0.0087	−0.0098	−0.0094	−0.0087	−0.0087	−0.0096	−0.0091	−0.0086	−0.0090	−0.0087	−0.0087
Point 25	0.5833	−0.1230	−0.1255	−0.1257	−0.1272	−0.1255	−0.1255	−0.1257	−0.1256	−0.1254	−0.1255	−0.1255	−0.1255
Point 26	0.5900	−0.2100	−0.2085	−0.2074	−0.2112	−0.2085	−0.2085	−0.2076	−0.2082	−0.2084	−0.2081	−0.2085	−0.2085

**Table 15:** The estimated current corresponding to the obtained best RMSE by the 11 algorithms on the DDM.

Case	$V_\text {m}$	$I_\text {m}$	$I_\text {e}$										
			EO	TSA	SOA	NNA	DE	MVO	WOA	BSA	CLNNA	MLNNA	ENNA
Point 1	−0.2057	0.7640	0.7640	0.7621	0.7716	0.7640	0.7640	0.7637	0.7624	0.7640	0.7643	0.7649	0.7640
Point 2	−0.1291	0.7620	0.7626	0.7611	0.7686	0.7626	0.7626	0.7625	0.7614	0.7626	0.7629	0.7632	0.7626
Point 3	−0.0588	0.7605	0.7614	0.7602	0.7658	0.7614	0.7613	0.7614	0.7606	0.7613	0.7617	0.7618	0.7613
Point 4	0.0057	0.7605	0.7602	0.7593	0.7633	0.7602	0.7602	0.7604	0.7598	0.7601	0.7605	0.7604	0.7602
Point 5	0.0646	0.7600	0.7591	0.7585	0.7609	0.7591	0.7591	0.7594	0.7591	0.7590	0.7594	0.7591	0.7591
Point 6	0.1185	0.7590	0.7581	0.7578	0.7588	0.7581	0.7581	0.7586	0.7585	0.7580	0.7585	0.7580	0.7581
Point 7	0.1678	0.7570	0.7572	0.7571	0.7568	0.7572	0.7572	0.7577	0.7578	0.7571	0.7576	0.7569	0.7572
Point 8	0.2132	0.7570	0.7562	0.7564	0.7549	0.7562	0.7562	0.7569	0.7571	0.7562	0.7566	0.7558	0.7562
Point 9	0.2545	0.7555	0.7552	0.7555	0.7531	0.7552	0.7552	0.7559	0.7563	0.7551	0.7555	0.7547	0.7552
Point 10	0.2924	0.7540	0.7537	0.7543	0.7509	0.7537	0.7537	0.7545	0.7550	0.7537	0.7541	0.7532	0.7537
Point 11	0.3269	0.7505	0.7514	0.7522	0.7481	0.7514	0.7514	0.7522	0.7528	0.7514	0.7517	0.7509	0.7514
Point 12	0.3585	0.7465	0.7473	0.7483	0.7436	0.7473	0.7473	0.7479	0.7486	0.7474	0.7474	0.7469	0.7473
Point 13	0.3873	0.7385	0.7400	0.7412	0.7362	0.7400	0.7400	0.7404	0.7410	0.7401	0.7399	0.7397	0.7400
Point 14	0.4137	0.7280	0.7272	0.7286	0.7233	0.7272	0.7273	0.7273	0.7277	0.7273	0.7269	0.7272	0.7272
Point 15	0.4373	0.7065	0.7069	0.7083	0.7028	0.7069	0.7069	0.7064	0.7067	0.7069	0.7061	0.7070	0.7069
Point 16	0.4590	0.6755	0.6752	0.6767	0.6709	0.6752	0.6752	0.6744	0.6745	0.6752	0.6742	0.6756	0.6752
Point 17	0.4784	0.6320	0.6308	0.6320	0.6258	0.6308	0.6308	0.6297	0.6296	0.6306	0.6297	0.6312	0.6308
Point 18	0.4960	0.5730	0.5720	0.5727	0.5662	0.5720	0.5720	0.5709	0.5707	0.5718	0.5710	0.5724	0.5720
Point 19	0.5119	0.4990	0.4997	0.4998	0.4930	0.4997	0.4997	0.4989	0.4987	0.4995	0.4991	0.5000	0.4997
Point 20	0.5265	0.4130	0.4137	0.4132	0.4063	0.4137	0.4137	0.4135	0.4134	0.4136	0.4136	0.4138	0.4137
Point 21	0.5398	0.3165	0.3175	0.3165	0.3099	0.3175	0.3175	0.3179	0.3179	0.3175	0.3179	0.3174	0.3175
Point 22	0.5521	0.2120	0.2121	0.2108	0.2052	0.2121	0.2121	0.2130	0.2131	0.2122	0.2129	0.2118	0.2121
Point 23	0.5633	0.1035	0.1021	0.1010	0.0971	0.1021	0.1022	0.1032	0.1034	0.1023	0.1030	0.1018	0.1022
Point 24	0.5736	−0.0100	−0.0088	−0.0091	−0.0105	−0.0088	−0.0088	−0.0082	−0.0080	−0.0087	−0.0082	−0.0090	−0.0088
Point 25	0.5833	−0.1230	−0.1255	−0.1246	−0.1227	−0.1255	−0.1255	−0.1257	−0.1256	−0.1255	−0.1256	−0.1255	−0.1255
Point 26	0.5900	−0.2100	−0.2083	−0.2059	−0.2010	−0.2083	−0.2084	−0.2098	−0.2098	−0.2085	−0.2093	−0.2080	−0.2084

**Table 16:** The estimated current corresponding to the obtained best RMSE by the 11 algorithms on the PVM.

Case	$V_\text {m}$	$I_\text {m}$	$I_\text {e}$										
			EO	TSA	SOA	NNA	DE	MVO	WOA	BSA	CLNNA	MLNNA	ENNA
Point 1	0.1248	1.0315	1.0295	1.0279	1.0278	1.0293	1.0291	1.0284	1.0292	1.0289	1.0295	1.0289	1.0291
Point 2	1.8093	1.0300	1.0277	1.0267	1.0269	1.0276	1.0274	1.0268	1.0277	1.0272	1.0278	1.0273	1.0274
Point 3	3.3511	1.0260	1.0259	1.0255	1.0260	1.0260	1.0257	1.0253	1.0262	1.0256	1.0261	1.0257	1.0257
Point 4	4.7622	1.0220	1.0241	1.0243	1.0250	1.0243	1.0241	1.0238	1.0247	1.0240	1.0245	1.0242	1.0241
Point 5	6.0538	1.0180	1.0222	1.0228	1.0237	1.0225	1.0223	1.0221	1.0230	1.0223	1.0226	1.0224	1.0223
Point 6	7.2364	1.0155	1.0198	1.0206	1.0218	1.0201	1.0199	1.0198	1.0207	1.0199	1.0202	1.0201	1.0199
Point 7	8.3189	1.0140	1.0162	1.0171	1.0184	1.0165	1.0164	1.0164	1.0171	1.0164	1.0166	1.0165	1.0164
Point 8	9.3097	1.0100	1.0103	1.0112	1.0126	1.0106	1.0105	1.0107	1.0112	1.0105	1.0106	1.0107	1.0105
Point 9	10.2163	1.0035	1.0004	1.0010	1.0024	1.0006	1.0006	1.0009	1.0010	1.0006	1.0006	1.0008	1.0006
Point 10	11.0449	0.9880	0.9844	0.9845	0.9858	0.9844	0.9845	0.9850	0.9846	0.9845	0.9843	0.9846	0.9845
Point 11	11.8018	0.9630	0.9595	0.9590	0.9601	0.9593	0.9595	0.9600	0.9591	0.9594	0.9591	0.9595	0.9595
Point 12	12.4929	0.9255	0.9229	0.9219	0.9226	0.9225	0.9228	0.9234	0.9220	0.9227	0.9223	0.9227	0.9228
Point 13	13.1231	0.8725	0.8727	0.8714	0.8716	0.8721	0.8726	0.8731	0.8714	0.8724	0.8719	0.8724	0.8726
Point 14	13.6983	0.8075	0.8074	0.8061	0.8057	0.8068	0.8073	0.8077	0.8058	0.8071	0.8066	0.8071	0.8073
Point 15	14.2221	0.7265	0.7285	0.7276	0.7264	0.7280	0.7283	0.7286	0.7268	0.7282	0.7278	0.7282	0.7283
Point 16	14.6995	0.6345	0.6373	0.6370	0.6353	0.6369	0.6371	0.6372	0.6358	0.6370	0.6368	0.6370	0.6371
Point 17	15.1346	0.5345	0.5363	0.5369	0.5346	0.5361	0.5362	0.5360	0.5352	0.5361	0.5361	0.5362	0.5362
Point 18	15.5311	0.4275	0.4295	0.4310	0.4283	0.4296	0.4295	0.4291	0.4289	0.4295	0.4296	0.4295	0.4295
Point 19	15.8929	0.3185	0.3187	0.3208	0.3181	0.3190	0.3188	0.3183	0.3186	0.3188	0.3191	0.3189	0.3188
Point 20	16.2229	0.2085	0.2073	0.2097	0.2072	0.2077	0.2074	0.2068	0.2075	0.2075	0.2078	0.2075	0.2074
Point 21	16.5241	0.1010	0.0960	0.0985	0.0963	0.0965	0.0962	0.0956	0.0966	0.0963	0.0966	0.0963	0.0962
Point 22	16.7987	−0.0080	−0.0084	−0.0066	−0.0081	−0.0081	−0.0083	−0.0087	−0.0078	−0.0083	−0.0080	−0.0082	−0.0083
Point 23	17.0499	−0.1110	−0.1110	−0.1100	−0.1107	−0.1108	−0.1109	−0.1110	−0.1104	−0.1109	−0.1108	−0.1109	−0.1109
Point 24	17.2793	−0.2090	−0.2092	−0.2095	−0.2092	−0.2094	−0.2092	−0.2090	−0.2088	−0.2093	−0.2094	−0.2093	−0.2092
Point 25	17.4885	−0.3030	−0.3007	−0.3027	−0.3012	−0.3013	−0.3009	−0.3002	−0.3007	−0.3011	−0.3015	−0.3010	−0.3009

**Table 17:** The optimal solutions obtained by ENNA and the reported algorithms on the SDM.

Algorithm	$I_\text {ph}(\text {A})$	$I_\text {sd}({\mu \text {A}})$	$R_\text {s}(\Omega )$	$R_\text {sh}(\Omega )$	*n*	$\text {RMSE}$
IJAYA	0.7608	0.3228	0.0364	53.7595	1.4811	9.8603E-04
JAYA	0.7608	0.3281	0.0364	54.9298	1.4828	9.8946E-04
GOTLBO	0.7608	0.3297	0.0363	53.3664	1.4833	9.8856E-04
CLPSO	0.7608	0.34302	0.0361	54.1965	1.4873	9.9633E-04
BLPSO	0.7607	0.36620	0.0359	60.2845	1.4939	1.0272E-03
GWO	0.760058925	0.3278	0.036776143	71.48825037	1.48240297	1.2803E-03
WDO	0.760831099	0.4283	0.035070711	55.85740062	1.5102104	1.2210E-03
IBSA	0.7607	0.35502	0.0361	58.2102	1.4907	1.0092E-03
LBSA	0.7606	0.34618	0.0362	59.0978	1.4881	1.0143E-03
LETLBO	0.7608	0.32597	0.0363	53.7429	1.4821	9.8738E-04
ENNA	0.7608	0.32302	0.03637709	53.718528	1.4811836	9.8602E-04

**Table 18:** The optimal solutions obtained by ENNA and the reported algorithms on the DDM.

Algorithm	$I_\text {ph}(\text {A})$	$I_\text {sd1}(\mu \text {A})$	$R_\text {s}(\Omega )$	$R_\text {sh}(\Omega )$	$n_1$	$I_\text {sd2}(\mu \text {A})$	$n_2$	$\text {RMSE}$
IJAYA	0.7601	0.0050445	0.0376	77.8519	1.2186	0.75094	1.6247	9.8293E-04
JAYA	0.7607	0.0060763	0.0364	52.6575	1.8436	0.31507	1.4788	9.8934E-04
GOTLBO	0.7608	0.13894	0.0365	53.4058	1.7254	0.26209	1.4658	9.8742E-04
CLPSO	0.7607	0.25843	0.0367	57.9422	1.4625	0.38615	1.9435	9.9894E-04
BLPSO	0.7608	0.27189	0.0366	61.1345	1.4674	0.43505	1.9662	1.0628E-03
GWO	0.7609	0.5099	0.0370	56.8758	1.9140	0.2161	1.4472	1.0270E-03
WDO	0.7608	0.2990	0.0354	44.6653	1.5443	0.1208	1.4551	1.681176E-03
IBSA	0.7608	0.21507	0.0366	51.9008	1.8718	0.26624	1.4651	9.9663E-04
LBSA	0.7606	0.29814	0.0363	60.1880	1.4760	0.27096	1.9202	1.0165E-03
LETLBO	0.7608	0.17390	0.0365	54.3021	1.6585	0.22664	1.4578	9.8565E-04
ENNA	0.7608	0.2259745	0.0367404	55.4854257	1.4510	0.74934	1.9999	9.8249E-04

**Table 19:** The optimal solutions obtained by ENNA and the reported algorithms on the PVM.

Algorithm	$I_\text {ph}(\text {A})$	$I_\text {sd}({\mu \text {A}})$	$R_\text {s}(\Omega )$	$R_\text {sh}(\Omega )$	*n*	$\text {RMSE}$
IJAYA	1.0305	3.4703	1.2016	977.3752	48.6298	2.425129E-03
JAYA	1.0302	3.4931	1.2014	1022.5	48.6531	2.427785E-03
GOTLBO	1.0307	3.5124	1.1995	969.9313	48.6766	2.426583E-03
CLPSO	1.0304	3.6131	1.1978	1017.0	48.7847	2.428064E-03
BLPSO	1.0305	3.5176	1.2002	992.7901	48.6815	2.425236E-03
GWO	1.029825039	4.3863	1.175731013	1186.592624	49.5468686	2.52608800172134E-03
WDO	1.029485112	4.0585	1.173311293	973.1519899	49.2486966	2.79601941233004E-03
IBSA	1.0305	3.4923	1.2010	986.7363	48.6537	2.425093E-03
LBSA	1.0304	3.5233	1.2014	1020.4	48.6866	2.429630E-03
LETLBO	1.0306	3.4705	1.2015	974.6190	48.6301	2.425116E-03
ENNA	1.0305	3.4823	1.2013	981.9822661	48.6428347	2.425075E-03

### Algorithm comparison

To investigate the performance of the proposed ENNA, it is compared with 10 powerful metaheuristic algorithms, which are EO^51^, TSA^52^, SOA^53^, NNA^34^, DE^54^, MVO^55^, WOA^56^, BSA^35^, CLNNA^57^, and MLNNA^58^. In addition, to make a fair comparison, the population size and maximum number of function evaluations are set to 30 and 45000 for all the applied algorithms, respectively. As presented in Section, ENNA does not need other control parameters. The other control parameters of the compared algorithms are extracted from the corresponding original references. Each algorithm is independently executed on each case 50 times.

Table 11, Table 12, and Table 13 present the statistical results of 13 algorithms in the SDM, DDM, and PVM, respectively. In the three tables, “MAX” means the worst value of 50 runs; “MEAN” means the mean value of 50 runs; “MEDIAN” means the median value of 50 runs; “MIN” means the best value of 50 runs; “STD” means the standard deviation of 50 runs. From Table 11, ENNA can get the best MAX, MEAN, MEDIAN, MIN, and STD. In addition, DE and ENNA can obtain the best MIN, i.e., 0.00098602. In addition, EO, NNA, and MLNNA also show excellent competitiveness, whose MIN are very close to those of ENNA and DE. In terms of MIN, WOA is 0.00119521; TSA is 0.00170553; SOA is 0.00284886. Clearly, WOA, TSA, and SOA are far inferior to the other algorithms, which are not suitable for solving the parameter extraction of the SDM. Although DE and ENNA can achieve the same MIN, in terms of MAX, MEAN, MEDIAN, and STD, ENNA is significantly superior to DE. That is, in terms of solution accuracy and stability, ENNA is the best of all the algorithms applied to the parameter extraction of the SDM. By carefully observing Table 12, ENNA is the best of all the applied algorithms in terms of the considered five indicators. The MIN obtained by ENNA is 0.000982485. In terms of MIN, EO, NNA, and DE are very close to ENNA. In addition, WOA, SOA, and TSA also show no competitive ability. The MIN of CLNNA and MLNNA are 0.001136820 and 0.001055564, respectively. Clearly, CLNNA and MLNNA cannot compete with ENNA. In general, ENNA shows obvious advantages in both solution quality and stability on the DDM. Looking at Table 13, ENNA can get the optimal MEAN and MIN. In terms of MEAN, ENNA is only inferior to TSA and MLNNA. Although TSA and MLNNA are better than ENNA on the MEAN, they cannot compete with ENNA on the MIN. DE and ENNA can achieve the optimal MIN, i.e., 0.00242507. EO, BSA, and MLNNA can get a similar MIN to those of DE and ENNA. Table 14, Table 15, and Table 16 show the estimated current of the applied algorithms according to the obtained MIN.

According to the estimated current in Table 14, Table 15, and Table 16, Fig. 13, Fig. 14, and Fig. 15 present the volt-ampere characteristic curve and volt-power characteristic curve obtained by ENNA in the SDM, DDM, and PVM, respectively. As can be seen from Fig. 13, Fig. 14, and Fig. 15, the experimental data obtained by ENNA are very similar to the benchmark data. This shows that the accuracy of the parameters estimated by ENNA is very high. At the same time, it also illustrates the excellent applicability of ENNA in solving photovoltaic model parameter estimation problems.

In order to further compare the performance differences between algorithms, the experimental results obtained by the algorithms are subjected to the Friedman test. Friedman test is a common method to compare the performance difference among algorithms by ranking, which has been used in many references^73–76^. Fig. 16 shows the ranking results from the Friedman test. From Fig. 16, all algorithms can be sorted in the following order from best to worst:

- SDM: ENNA, BSA, DE, EO, MLNNA, CLNNA, NNA, MVO, WOA, TSA, and SOA.
- DDM: ENNA, DE, BSA, EO, CLNNA, NNA, MLNNA, MVO, TSA, WOA, and SOA.
- PVM: ENNA, DE, BSA, EO, MLNNA, NNA, MVO, TSA, CLNNA, WOA, and SOA.

Clearly, ENNA is the best of all the applied algorithms in the three considered cases, which proves the excellent global search ability. In addition, DE, EO, and BSA also show strong stability, which are ranked between second and fourth in the three considered cases. SOA is the three worst algorithm.

To compare the convergence performance among different algorithms, Fig. 17, Fig. 18, and Fig. 19 show the convergence curves of ENNA and the compared algorithms in SDM, DDM, and PVM, respectively. From Fig. 17, SOA is the worst of all algorithms. WOA, and TSA quickly converge to a local optimum. Although CLNNA, NNA, MLNNA, EO, DE, and MVO have better convergence performance than SOA, WOA, and TSA, they are inferior to BSA and ENNA in terms of convergence performance. BSA has better convergence performance than EO, TSA, SOA, NNA, DE, MVO, WOA, CLNNA, and MLNNA, which still compete with ENNA. From Fig. 18, SOA is the worst of all algorithms. WOA, and TSA quickly exhibit the phenomenon of premature convergence. Although CLNNA, NNA, MLNNA, EO, BSA, and MVO have better convergence performance than SOA, WOA, and TSA, they are inferior to DE and ENNA in terms of convergence performance. DE shows better convergence performance than EO, TSA, SOA, NNA, BSA, MVO, WOA, CLNNA, and MLNNA, which is inferior to ENNA. From Fig. 19, SOA is the worst of all algorithms. CLNNA and WOA quickly exhibit the phenomenon of premature convergence. Although TSA, NNA, DE, MVO, BSA, and ENNA outperform SOA, WOA, and CLNNA, they have obvious disadvantages compared with MLNNA in terms of convergence performance. Note that, although the convergence performance of ENNA is not the best, it is second only to MLNNA and TSA. Therefore, in summary, ENNA demonstrates highly competitive convergence performance.

Table 17, Table 18, and Table 19 compare the optimal solutions obtained by ENNA and the reported algorithms. Specifically, the solutions of the reported algorithms in the three tables are extracted from^61,72^. From Table 17, the optimal RMSE of ENNA is 9.8602E-04, which is the best of all algorithms. The optimal RMSE of IJAYA is 9.8603E-04, which is very similar to that of ENNA. In addition, the optimal RMSEs of BLPSO, GWO, WDO, IBSA, and LBSA are 1.0272E-03, 1.2803E-03, 1.2210E-03, 1.0092E-03, and 1.0143E-03, which are far more than that of ENNA. That is, in terms of accuracy in estimating parameters on the SDM, ENNA is far superior to BLPSO, GWO, WDO, IBSA, and LBSA. Looking at Table 18, the optimal RMSE achieved by ENNA is 9.8249E-04, which is the best of all algorithms. The optimal RMSE of IJAYA is 9.8293E-04, which is very close to that of ENNA. In addition, the optimal RMSE of BLPSO, GWO, WDO, and LBSA are 1.0628E-03, 1.0270E-03, 1.681176E-03, and 1.0165E-03, respectively. In other words, the accuracy of the parameters on the DDM estimated by BLPSO, GWO, WDO, and LBSA is far inferior to that of ENNA. As can be seen from Table 19, the optimal RMSE of ENNA is 2.425075E-03, which outperforms those of the other algorithms. The optimal RMSEs of IJAYA, IBSA, and LETLBO are 2.425129E-03, 2.425093E-03, and 2.425116E-03, respectively. That is, IJAYA, IBSA, and LETLBO have demonstrated strong competitiveness. Note that the optimal RMSEs of GWO and WDO are 2.52608800172134E-03 and 2.79601941233004E-03, respectively. Clearly, in terms of parameter estimation accuracy on the PVM, GWO and WDO are significantly inferiorthe to the other algorithms.

### Discussion on the validity of the improved strategies

The proposed ENNA is an improved version of NNA. ENNA is designed based on two defined operators, i.e., the perturbation operator and elite operator. The function of the perturbation operator is to increase the perturbation for the designed three transfer strategies. The function of the elite operator is to design the learning strategy in the transfer operator. The two operators can enhance ENNA’s ability to escape from the local optimal solutions. In addition, in ENNA, the bias operator and transfer operator have the same chance to be executed, which can maximize the advantages of the bias operator and transfer operator.

As shown in Table 11, ENNA is significantly better than NNA, CLNNA, and ENNA in MAX, MEAN, MEDIAN, and STD. In addition, although the MIN of NNA and MLNNA are 0.00098619 and 0.00098728, respectively, they cannot compete with ENNA (the MIN of ENNA is 0.000098602). Note that the MIN of CLNNA is 0.00102053, which is significantly inferior to that of ENNA. Looking at Table 12, ENNA has a very obvious advantage over NNA, CLNNA, and MLNNA. Specifically, in terms of MIN, NNA, CLNNA, MLNNA, and ENNA are 0.000984631, 0.001136820, 0.001055564, and 0.000982485, respectively. From Table 13, in terms of MIN, NNA, CLNNA, MLNNA, and ENNA are 0.00243953, 0.00245611, 0.00242828, and 0.00242507, respectively. Clearly, NNA, CLNNA, MLNNA, and ENNA are very similar. However, ENNA is slightly better than NNA, CLNNA, and MLNNA. From Fig. 13, ENNA is the best of all the applied algorithms in the three considered cases. However, NNA is inferior to ENNA, BSA, DE, EO, MLNNA, and CLNNA on the SDM; MLNNA is inferior to ENNA, BSA, DE, and EO on the SDM; CLNNA is inferior to ENNA, BSA, DE, EO, and MLNNA on the SDM; NNA cannot compete with ENNA, DE, BSA, EO, and CLNNA on the DDM; MLNNA cannot compete with ENNA, DE, BSA, EO, CLNNA, and NNA on the DDM; CLNNA cannot compete with ENNA, DE, BSA, and EO on the DDM; ENNA, DE, BSA, EO, and MLNNA are superior to NNA on the PVM; ENNA, DE, BSA, and EO are superior to MLNNA on the PVM; ENNA, DE, BSA, EO, MLNNA, NNA, MVO, TSA, and CLNNA are superior to CLNNA on the PVM.

Based on the above discussion, ENNA’s overall performance is superior to that of NNA, MLNNA, and CLNNA. That is, the improved strategies in ENNA are very effective, which can significantly improve the ability of ENNA to escape local optima.

## Conclusion

As a clean energy with broad application prospects, the efficient use of solar energy is of vital importance. The PV system is a key link in converting solar energy into electrical energy. Its conversion efficiency directly determines the utilization rate of solar energy. Therefore, optimizing, controlling and simulating the photovoltaic system are the key approaches to enhance its energy conversion efficiency. The foundation for achieving all this lies in how to accurately extract the unknown parameters in the PV model. This paper proposes a new population-based metaheuristic algorithm, called ENNA, for the parameter estimation of PV models. ENNA is a variant of NNA, whose main mechanism is based on the defined perturbation operator and elite operator. Specifically, the perturbation operator is based on a random number obeying the standard normal distribution; the elite operator is based on the mean position of the population and the crossover matrix. According to the two operators, three learning strategies in the transfer operator are designed, which refer to the mean position of the population, the optimal position of the population, and the historical population. In addition, a random number obeying the uniform distribution between 0 and 1 is used to balance the execution time of the bias operator and transfer operator. Note that the improved strategies do not introduce any other control parameters. To investigate the performance of ENNA, ENNA is employed to solve the parameter extraction of three different types of PV models, i.e., SDM, DDM, and PVM. Experimental results show that ENNA outperforms the compared algorithms, i.e., EO, TSA, SOA, NNA, DE, MVO, WOA, BSA, CLNNA, and MLNNA, in terms of parameter estimation accuracy. This indicates that ENNA demonstrates extremely outstanding performance in extracting unknown parameters of PV models.

According to the obtained experimental results, the strength and weakness of ENNA can be described as follows. The main idea of the proposed ENNA is based on the defined perturbation operator and elite operator. Specifically, the perturbation operator is based on a random number obeying the standard normal distribution; the elite operator is based on the mean position of the population and the crossover matrix. According to the two operators, three learning strategies in the transfer operator are designed, which refer to the mean position of the population, the optimal position of the population, and the historical population. In addition, a random number obeying the uniform distribution between 0 and 1 is used to balance the execution time of the bias operator and transfer operator. Therefore, the strength of ENNA is its excellent global search capability. In addition, it is worth mentioning that ENNA does not demonstrate significant advantages in computational efficiency for the following reasons. Like NNA, ENNA also implements algorithm design through the architecture of feedback type artificial neural networks, and the computation of neurons in neural networks significantly increases computational overhead. As the population size increases, the increase in computational overhead becomes even more significant. Therefore, how to improve the computational efficiency of ENNA is also an important direction for future research. We plan to improve the computational efficiency of ENNA by combining dropout technology with reinforcement learning in the future.

The proposed ENNA can be conveniently applied to different types of engineering optimization problems with only the required population size and maximum number of iterations. Therefore, in future research, we will attempt to use ENNA to solve more engineering optimization problems, such as the optimal scheduling in energy hub considering battery lifetime, the ultrasonic guided wave multi-damage localization, the functional brain network classification, and the optimal scheduling of electric vehicle ordered charging and discharging.

## Data Availability

The data and materials used to support the findings of this study are available from the corresponding author upon reasonable request.
